# Sensor-Driven World Models for Embodied Intelligence: A Survey of Sensor–State–Decision Modeling Across Far-Field and Near-Field Regimes

**DOI:** 10.3390/s26185944

**Published:** 2026-09-19

**Authors:** Yi Yang, Hao Feng, Beichen Lu

**Affiliations:** Department of Electronic Engineering, Tsinghua University, Beijing 100084, Chinalubc23@mails.tsinghua.edu.cn (B.L.)

**Keywords:** world models, embodied intelligence, sensor–state–decision framework, far-field and near-field modeling, long-tail scenarios, physical intelligence

## Abstract

World models are becoming a crucial foundation for embodied intelligence, which transforms sensor observations into internal state representations that support prediction, planning, and control. However, existing research is scattered across different fields (autonomous driving, robotic manipulation, generative interactive environments, and physical intelligence), and lacks a description of the role of sensors in defining the observable world, shaping state representations, and influencing decision-making. This paper proposes a unified Sensor–State–Decision (SSD) framework to describe sensor-driven world models. Specifically, the Sensor-Layer acquires raw physical observation data, the State-Layer transforms it into a structured world state representation, and the Decision-Layer outputs the final executable decision. Furthermore, it proposes that the far-field and near-field mechanisms describe the relative positions between complete SSD instances in the SSD hierarchy. That is, higher layers constrain lower layers through goal injection, and lower layers correct higher layers through result feedback. Based on this framework, we synthesize representative architectures, applications, datasets, and benchmarks, particularly long-tail scenarios in the Far-Field and Near-Field domains. Finally, we summarize the current challenges. The SSD perspective promises to provide a new structured analytical framework for designing robust, physically based, and deployable embodied world models in the real world.

## 1. Introduction

World Models are a buzzword in cutting-edge fields like artificial intelligence and embodied intelligence, considered a pathway to spatial intelligence. However, in different applications, such as video generation or physics engines, World Models have entirely different roles and structures. Compared to large language models, the physical world needs World Models to express temporal and spatial cues.

One of the landmark papers on world models was published in 2018 by David Ha et al. [1], which proposed the basic structure of modern world models and pointed out that agents can be trained in their own hallucinated dreams. In 2019, PlaNet [2], proposed by Danijar Hafner et al., proved that world models can be planned in a more compact latent space, rather than in pixel space. Subsequently, they proposed Dreamer [3] as a reinforcement learning agent, further transforming PlaNet’s “planning” into latent imagination—that is, imagining in latent space—and using imagined data to train the policy. Then, in 2023, DreamerV3 [4] was proposed, achieving stable operation on over 150 different tasks using the same world model, taking another step towards becoming a general-purpose intelligent agent algorithm framework. The MuZero algorithm [5], published in Nature in 2020, proposes that it does not need to learn all the details of the complete world, but only the reward, value, and policy that are most useful for planning. Mahmoud Medany et al. utilize Dreamer v3 to autonomously control an ultrasonically driven microrobot and trained an actor-critic architecture in an imagined model [6]. In the field of autonomous driving, Y Guan et al. adopt a world model to augment accident-related driving data, especially complex interactions, long-tail scenarios, and changes in visual conditions, to obtain a spatiotemporal risk prediction model [7]. Proposed in 2025, the WHAM [8] is a world model learning from complex 3D games and actions of real human players. It generates user-modifiable gameplay sequences based on approximately 500,000 game session data for game ideation. Genie [9] was introduced by DeepMind in 2024, which was a generative interactive environment trained unsupervised on a vast collection of web videos without action labels, resulting in the creation of action-controllable virtual worlds. A Nature News Explainer published in April 2026 pointed out that training AI world models with physical environment data could improve the real-world capabilities of technologies such as robotics. It argues that world models represent the next stage of AI development, moving beyond statistical learning from text and images on the internet to modeling, predicting and acting upon the physical world [10]. As these studies demonstrate, world models have currently emerged as a primary framework for modeling, prediction, and action in both the physical and 3D worlds, representing an effective pathway for building spatial intelligence in these domains.

Our research indicates that prior to 2023, terms such as pixels, images, observations, and states frequently appeared in world model papers, while “sensors” was rarely a core term; however, since 2023—particularly with the rise of physical AI, autonomous driving, and robotics—there has been a proliferation of terms related to sensors or sensor modalities, such as camera, LiDAR, RGB-D, proprioception, action, audio, and multi-sensor outputs. Table 1 provides a concise comparison of this transition across different categories of world model research.

Early sensors were hardware devices that passively captured information from the physical world. However, recent research indicates a shift in the role of sensors within world models: they are evolving from mere data sources into the foundation for modeling physical states in both the physical and 3D worlds. While early world model research often abstracted visual frames, pixels, or environmental observations into a generalized “observation,” the past three years have seen sensors—such as cameras, LiDAR, RGB-D, proprioception, action states, and audio—become integral elements of world model training and inference. These sensors provide real-world state information, enforce physical consistency, supervise future predictions, and enable closed-loop verification of action consequences within both physical and 3D environments.

While sensors have become increasingly important in recent world model research, their roles differ across applications such as physical AI, autonomous driving, and robotics. Table 2 summarizes a concise comparison of the main roles and functions of the sensors in these specific fields.

In robotic world models, sensors not only provide foundational visual information but also offer a unified representation of the embodied robot’s state, its contact dynamics, and the consequences of its subsequent actions. For instance, PointWorld [15] unifies robot actions into a 3D spatial representation using visual observations and action commands. DreamZero [16] jointly models video and action data streams to achieve generalization across tasks, environments, and robot embodiments. Meanwhile, Visuo-Tactile World Models [19] incorporate contact dynamics to ensure physical realism in scenarios involving visual occlusion. In autonomous driving world models, multiple sensors are employed to provide finer-grained environmental perception and to simulate long-tail events. For instance, MUVO [23] learns sensor-agnostic spatial representations from camera and LiDAR data to predict the multimodal data required by the world model. UniDriveDreamer [25] unifies multi-camera and LiDAR data into a single-stage multimodal world model. Cosmos-Drive-Dreams [26] leverages world models to generate long-tail driving scenarios and provide corresponding labels. In generative interactive environments, world models have evolved—through sensor-based observation—from simple video generators into interactive world models that undergo continuous correction and maintain spatiotemporal consistency. For instance, Genie [9] learns latent actions from video frames to enable frame-by-frame control of a generative virtual world. UniSim [14] constructs an interactive world simulator by integrating heterogeneous visual and action data to predict visual evolution. WHAM [8] learns gameplay dynamics using game footage and controller actions. In physical AI, sensors in video, motion, and physical scenes are responsible for transmitting data to digital twins and world models: visual sensors provide the world state flow for autonomous vehicles/humanoid robots, actions serve as intervention signals, and trajectory sensors provide the consequences of actions, enabling the world model to learn the current state and actions and generate future states and actions, especially for generating closed-loop control of states and action generation in synthetic and simulated long-tailed scenarios [16,18].

These advancements demonstrate that the core value of world models lies not only in the compression of sensor data, but also in obtaining the internal states for the next steps of prediction, planning, and control based on sensor observations. The goal is to transform the real physical world into states and actions within a trainable, simulable, and verifiable multimodal world model.

This survey is motivated by the fact that embodied intelligent agents, such as autonomous vehicles/humanoid robots, cannot truly observe the world directly like humans. Embodied intelligent agents rely on the stable transformation of multimodal sensor data streams into internal states to decide on their next actions. Autonomous vehicles rely on cameras, LiDAR, radar, GNSS/IMU, and their own motion signals to infer traffic scenes, dynamic objects, road structures, and future trajectories. In contrast, humanoid robots rely on cameras, force sensing, and tactile feedback to infer environmental interactions, contact dynamics, object availability, and interaction outcomes. Therefore, for sensor researchers, the key question is: how could multiple heterogeneous sensor physical data be directly or indirectly used to serve the state and actions in multimodal world models? Specifically, how could the states in long-tail driving and long-tail operation scenarios be generated more realistically through synthesis and simulation, such as accident-related driving data, and sparse, occluded, uncertain, and cross-modal robot states?

Another challenge for sensor researchers is that, due to the inherent physical limitations of sensor devices, such as signal-to-noise ratio, field of view, and spatial resolution, a single sensor often faces a trade-off between sensing distance and detail resolution, making it impossible to achieve both long-range environmental perception and close-range fine observation. A key challenge is how to delineate the interface between far-field and near-field data from heterogeneous multi-sensor data for specific application scenarios. For example, the speed of autonomous vehicles necessitates that far-field sensors detect risks in advance. Near-field sensors handle detailed processing of scenarios such as traffic jams, parking, and lane changes. However, in robotics scenarios, the distinction between far-field and near-field is not based on absolute distance but rather on whether the object has entered the operable space. For instance, the far-field answers “Is the cup on the left or right side of the table?”, while the near-field answers “Has the gripper touched the cup?”.

Existing research and surveys have provided valuable insights on reinforcement learning, generative world models, autonomous driving prediction, robot manipulation, multimodal perception, and vision–language–action systems. However, these publications typically focus on algorithms, application domains, or model families, rather than the complete sensor-to-decision path. Furthermore, the same terms such as state, prediction, planning, and world representation often have different definitions in different contexts. Currently, there is a lack of a unified explanation of how multimodal sensor perception, proprioceptive integration, tactile integration, state representation, world model architecture, and decision-oriented behavior collectively shape far-field and near-field embodied world models.

These observations prompted us to conduct a sensor-centric survey: Starting with heterogeneous multi-sensor physical data from world models, this paper systematically reviews the construction mechanisms of physical scene state representations; further, it discusses how far-field environmental perception and near-field fine observation could be unified to serve scene reconstruction and synthetic simulation, and explores how these state representations can be transformed into decision-oriented behavior generation, paying particular attention to the challenges introduced by sparse observations, occlusion, uncertainty, and cross-modal approaches in long-tailed scenes.

The main contributions of this survey are as follows.

- We reveal the increasingly important role of sensors in world model research. Sensors have evolved from early passive hardware devices for collecting physical information to key components for state modeling, physical consistency, closed-loop verification, and future prediction. Furthermore, this survey proposes that world models need to utilize heterogeneous sensor data to construct states in sparse, occluded, uncertain, and cross-modal long-tailed scenarios for scene reconstruction, synthetic simulation, future prediction, and behavior generation.
- We propose a unified Sensor–State–Decision (SSD) framework. To provide a common structure for comparing different world model architectures across domains, this survey proposes that the world model architecture needs to connect three key layers: a physical sensor layer, defining the observation boundaries of embodied agents; a state layer, completing world modeling and internal representation; and a decision layer, translating representations into planning, control, and behavior.
- We introduce far-field and near-field perspectives. By considering the difference between perception distance and spatial resolution, this survey proposes the need for an interface between far-field perception for global scene understanding and long-term temporal prediction, and near-field perception for fine-grained interaction, contact dynamics, and local physics.

The remainder of the paper is organized as follows. Section 2 introduces the methodology and scope of this survey. Section 3 defines the conceptual boundary of world models. Section 4 introduces the SSD framework, the far-field and near-field dimension, and the recursive structure that joins them. Section 5, Section 6 and Section 7 analyze the Sensor, State, and Decision layers in detail. Section 8 is the architecture survey, and Section 9 examines the application domains. Section 10 discusses datasets and benchmarks. Section 11 summarizes the open challenges and research directions. Section 12 concludes the paper and describes future perspectives.

## 2. Survey Methodology and Scope

In terms of literature classification, this survey does not proceed solely by model or application but instead uses the Sensor–State–Decision (SSD) approach. This survey focuses on (1) physical sensor observation information; (2) this information being encoded into world-state representations; and (3) these representations being decoded into final decisions. The literature primarily comes from world-model literature on autonomous driving, robotics, generative interaction, and physically embodied systems. Autonomous driving and robotics are considered more extensively because these two systems can be seen as the most straightforward physical foundational examples of the Sensor–State–Decision approach. Furthermore, autonomous driving provides multi-agent interaction and safety-oriented far-field multimodal perception systems; surgical robots provide near-field contact systems requiring high-frequency control. Therefore, these areas are used as two case studies for analyzing recursive SSD instances. Other case studies include generative interactive environments and physical AI platforms, providing cross-domain world model simulation and state generation.

This literature search aimed to cover the foundations of world models before 2023 and sensor-based world models after 2023. The primary search period was the latest literature available from January 2018 to the time of manuscript revision. The relevant literature came mainly from Web of Science, Scopus, IEEE Xplore, ACM Digital Library, and Google Scholar, as well as arXiv and publicly available technical reports. The search used terminology combinations from four query groups:

1. World model, predictive world model, latent dynamics, embodied intelligence, embodied AI, physical AI, spatial intelligence, and interactive world model.
2. Autonomous driving, robotics, robot manipulation, surgical robotics, humanoid robot, interactive generative environment, world simulator, and digital twin.
3. Sensor, multimodal sensor, camera, LiDAR, radar, RGB-D, proprioception, tactile, force, state representation, world state, latent state, occupancy, BEV, and 3D representation.
4. Prediction, action-conditioned prediction, planning, control, policy, closed-loop, long-tail, occlusion, uncertainty, sensor failure, far-field, near-field, hierarchical control, and multiscale modeling.

Literature was selected based on its relevance to the sensor–state–decision path, rather than solely on the presence of terms such as “world model” or “multimodal”. The selection of datasets and benchmarks primarily considers whether they support one or more attributes relevant to embodied world modeling. For high-value research, datasets, papers, etc., discovered in the initial search, forward and backward reference tracking was also employed; early literature typically uses terms such as “observation, pixel, image, and latent state,” while recent literature often uses terms such as “camera, LiDAR, RGB-D, proprioception, touch, force, and audio”; the reference lists of relevant surveys were consulted to retrieve more possible literature. This survey prioritizes peer-reviewed journal and conference papers. Preprints and industry reports are primarily used to document the latest research, with efforts made to ensure consistency with the latest versions. To ensure a fair comparison of various research findings, this survey provides a methodology organized according to the sensor–state–decision path, as shown in Table 3. The same dimensions will be used for subsequent comparisons.

## 3. World Model Concepts and Positioning

### 3.1. World Model Definition

A world model is an internal, learnable representation that enables an agent to simulate its environment, predict the consequences of actions, and reason about future states without physical interaction. This notion has its intellectual roots in cognitive science and was formally instantiated in deep learning by Ha and Schmidhuber [1], whose architecture demonstrated that agents could be trained entirely within hallucinated dream environments and subsequently transfer policies to the real world. Modern formulations have substantially broadened this foundation: DreamerV3 [4] showed that a single algorithm with fixed hyperparameters could master over 150 tasks spanning continuous control, visual reasoning, and open-world game-playing, while LeCun [30] proposed the Joint-Embedding Predictive Architecture (JEPA), shifting emphasis from pixel-level reconstruction to prediction in abstract representation space to avoid modeling unpredictable environmental noise. Currently, World Models can be functionally categorized as Renderers, Simulators, and Planners, and representation as pixel/video, abstract latent variables, explicit 3D states, and object-relationship-causal graphs.

From a fundamental research perspective, two research programs anchor the field: Yann LeCun’s abstract prediction approach [13,30] and Fei-Fei Li’s spatial intelligence approach. One pathway of abstract prediction is to utilize masked joint embedding to predict abstract continuous embeddings. V-JEPA 2.1 [12] is the latest advancement in the JEPA approach. Unlike pixel-level generation methods, V-JEPA learns dense predictive loss, deep self-supervision, multimodal tokenizers, and model/data scaling, allowing the model to simultaneously gain global scene understanding and sufficiently fine-grained spatiotemporal representations.

The spatial intelligence program, in turn, extracts world models with spatial structure from multimodal data in order to reconstruct and simulate 3D space, with lineage in large-scale visual grounding efforts. A recent instantiation of this route, the PointWorld model [15] in 2026, contains approximately 2 million trajectories and 500 h of training data from real and simulated robot maneuvers. By constructing an extension of the 3D world model, multiple manipulation behaviors can be implemented on physical hardware using only a single actual RGB-D image.

Papers published in the last three years have mainly expanded the world model from traditional reinforcement learning to four directions: robotics, autonomous driving, generative interactive environments, and physical AI. From the perspective of engineering applications such as robotics and autonomous driving, NVIDIA’s Cosmos World Foundation Model (WFM) approach proposes building digital twin models of the physical world using large-scale video training datasets. Cosmos WFM builds diffusion and autoregressive transformer models using continuous and discrete latent representations [28].

In 2026, Nvidia’s Cosmos 3 [29] is a family of multimodal world models that unifies visual language models, video generators, world simulators, and world action models, enabling joint processing and generation of language, images, videos, audio, and action sequences. Meanwhile, as a World Action Model (WAM), DreamZero [16] could achieve few-shot embodiment adaptation with 30 min of play data while maintaining zero-shot generalization. As a synthetic data pipeline, DreamGen [17] leverages video world models to create realistic training data applicable to various robots, environments and tasks. GR00T N1 [18] is an open foundational model for general-purpose humanoid robots. It combines a visual language understanding model and a diffusion-based motion generation model for end-to-end joint training. GR00T N1 belongs to the Vision–Language–Action (VLA) model.

From the perspective of large-scale training of generated videos, OpenAI’s Sora and Google DeepMind’s Genie [9] employ extended video generation models and latent action models, respectively. Both use massive amounts of unlabeled video on the internet as training data for unsupervised training, enabling AI agents to learn more behaviors that exist in the latent action space that are not included in the training data.

From the perspective of applications in the autonomous driving industry, Wayve’s GAIA [20] route combines data from multiple cameras, vehicle status, route information, and other traffic participants to form a shared representation. An autoregressive Transformer (predicts the next token of the image) and a diffusion decoder (restores the pixel space) are utilized to achieve the goal of generating a world model and evaluating driving scenarios.

### 3.2. Distinguishing World Models from Related Paradigms

Given the proliferation of related terms in the literature, it is necessary to establish precise conceptual boundaries.

Perception models such as BEVFormer [31] map sensor observations to semantic representations in a purely feedforward manner, producing outputs that are retrospective rather than anticipatory. They describe what the world currently looks like, not how it will evolve. Generative models such as Stable Diffusion and DALL-E produce high-fidelity samples from a learned data distribution. DALL-E conditions image generation on text embeddings derived from a hierarchical diffusion process, while Stable Diffusion operates in a compressed latent space. Despite their architectural differences, neither model is conditioned on explicit agent actions nor does it model the causal structure of environment dynamics. LLMs and Vision–Language–Action (VLA) models such as GPT-4 [32] and RT-2 [33] exhibit impressive semantic reasoning and can output plausible action sequences. OpenVLA [34] extends this paradigm by fine-tuning a vision-language backbone on robot demonstration data to directly predict motor actions. However, such models treat actions fundamentally as language-space outputs derived from reasoning rather than as inputs that drive forward simulation of world state. Concurrent work in other LLM-driven applications reinforces the contrast: multi-agent frameworks that use adversarial debate and voting to suppress LLM hallucinations [35], LLM-annotated pipelines for depression detection on social media [36], and LLM-agent recommender systems that bridge behavior and semantic signals [37] all exploit language reasoning on static or batch inputs. They do not maintain a transition model that propagates state under action, nor do they expose a closed-loop, action-conditioned update of a future observation. Nor is the bottleneck purely algorithmic: the engineering of LLM inference itself, including prefill-only optimization tailored to discriminative and single-token-decision workloads [38], sits outside the sensor–state–decision path, even when those single-token decisions are eventually consumed inside an embodied control loop. Consequently, as Zhu et al. [39] observe, internet-scale vision-language pre-training substantially improves semantic generalization but does not endow models with the closed-loop, action-conditioned prediction required for counterfactual reasoning and look-ahead planning.

World models, by contrast, are distinguished by three defining characteristics. They accept sensor observations and agent actions as joint inputs, maintain an explicit representation of world state that evolves under learned dynamics, and ensure that this state representation is decision-relevant for downstream prediction, planning, and control.

Therefore, a complete framework is needed to process heterogeneous sensor data in autonomous driving, robotic operations, and physical AI systems, enabling cross-platform migration capabilities. This means extracting universal physical laws such as occlusion, collision, and friction implicit in heterogeneous sensor data, forming a multi-layered representation shared across different applications. This representation ranges from the underlying raw sensor features to higher-level semantics, causality, and underlying physical laws, primarily preserving the high-level physical and semantic representations during cross-platform migration. Different models acquire the same explanation of “what the physical object is, how it moves, and how it interacts,” regardless of “which type of sensor does the data come from?”.

## 4. The Sensor–State–Decision Framework

### 4.1. Sensor–State–Decision Framework

World models are not defined only by their ability to predict future observations. For embodied intelligence, their central role is to connect sensor-grounded perception with representations that can support planning, control, and other decisions. Existing formulations, including World Models [1], Dreamer [3], and predictive architectures for autonomous intelligence [30], establish this observation-state-action connection, but they usually leave the sensor interface implicit. As a result, they do not fully specify how sensing modalities set the information boundary, how observations become decision-relevant state, or how uncertainty should move through the model.

**Figure 1:**
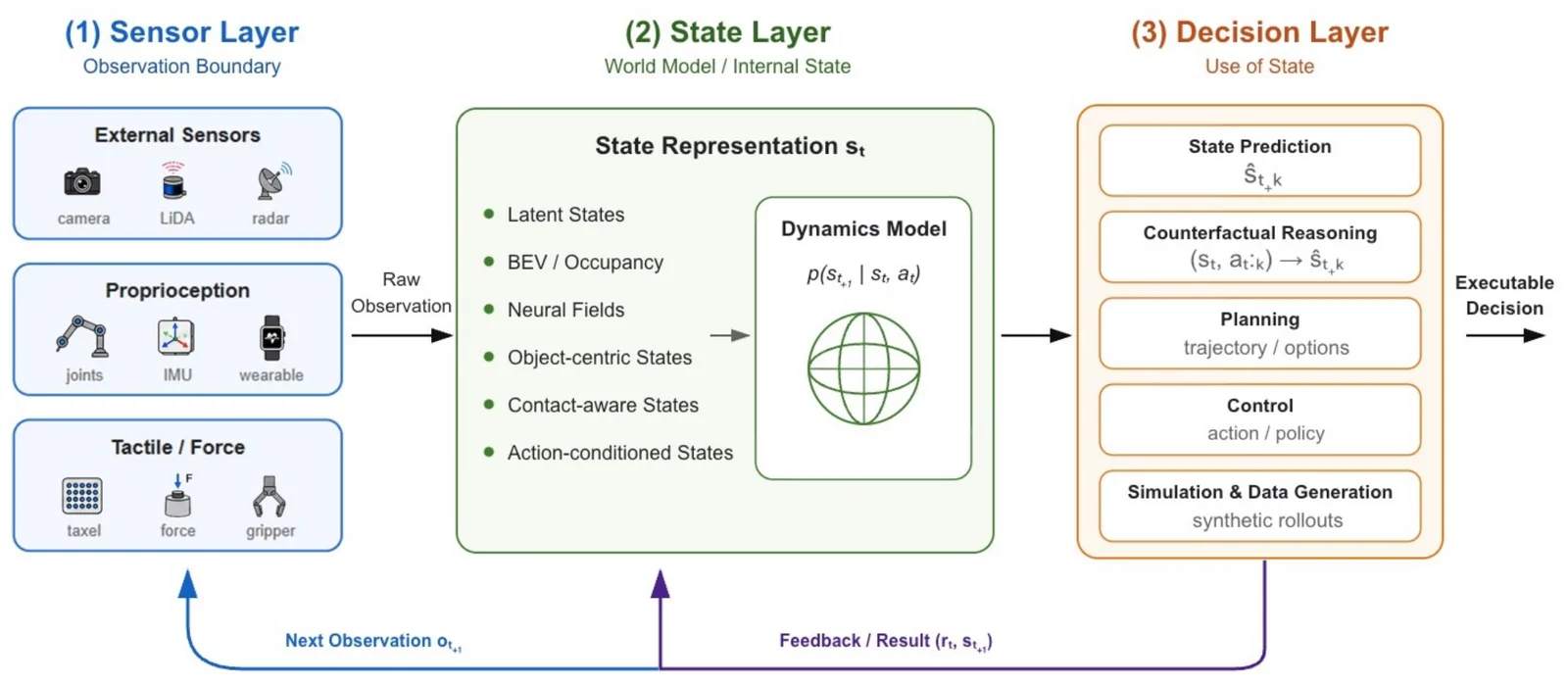
Single-level Sensor–State–Decision loop. The Sensor Layer defines the observation boundary, the State Layer constructs and predicts an internal world state, and the Decision Layer uses that state for prediction, planning, control, and other executable outputs. Executed decisions generate feedback and subsequent observations, closing the loop.

We therefore organize sensor-driven world models through a Sensor–State–Decision (SSD) framework. The Sensor Layer acquires physical, simulated, or data-derived observations; the State Layer converts current and historical observations into an explicit or implicit world state; and the Decision Layer uses current or predicted states to produce actions, plans, policies, trajectories, interventions, or decision support. Figure 1 shows the single-level loop. The three layers are mutually constrained: sensors determine what can be observed, the state layer determines what is retained and predicted, and the decision layer determines which state variables are operationally valuable.

### 4.2. Operational Taxonomy and Classification Rules

For SSD to function as a reusable taxonomy, each layer must be assigned by observable evidence in a paper or system rather than by intuition. Table 4 defines the minimum evidence and the fields that should be recorded when mapping a system.

We apply five classification rules. First, the Sensor Layer requires an identifiable observation source; methods using pre-generated video, latent tokens, or synthetic rollouts should still record sensor provenance, or else mark the layer as “not explicit”. Second, a feature qualifies as State only if it supports temporal prediction, environment representation, or decision-conditioned inference. Third, Decision requires a closed-loop environmental effect or an explicit action, trajectory, policy, planning, or intervention interface; pure prediction systems are marked as having “no explicit decision interface”. Fourth, transition and prediction models are State substructures when they roll the state forward, and cross-layer interfaces when their outputs are directly consumed by planning or control. Fifth, missing or implicit layers should remain missing or implicit rather than being forced into a complete three-layer diagram.

Table 5 gives representative and ambiguous mappings. Its purpose is not coverage, but repeatability: the same fields distinguish sensor provenance, state abstraction, transition model, decision interface, uncertainty treatment, and evaluation setting across systems. The ambiguous cases are especially important: camera-centric generators, occupancy forecasters, internet-video models, and visuo-tactile predictors should not be forced into the same decision-interface category simply because each predicts future states or observations.

SSD is adjacent to, but not identical with, several established frameworks. Table 6 summarizes the difference.

Table 7 fixes the operational terminology used in the remainder of the review.

### 4.3. Recursive Far-Field and Near-Field Structure

The far-field/near-field distinction is not triggered by a single distance threshold. For an SSD level *ℓ*, let $\lambda_{\ell }$ denote characteristic task-relevant spatial extent and $\tau_{\ell }$ denote characteristic update or decision timescale. Both are domain-dependent descriptors. Adjacent SSD levels can therefore be read as relatively far-field and near-field when their operating profiles differ across range, resolution, uncertainty, horizon, controllability, contact, and control frequency, even inside the same physical workspace.

**Figure 2:**
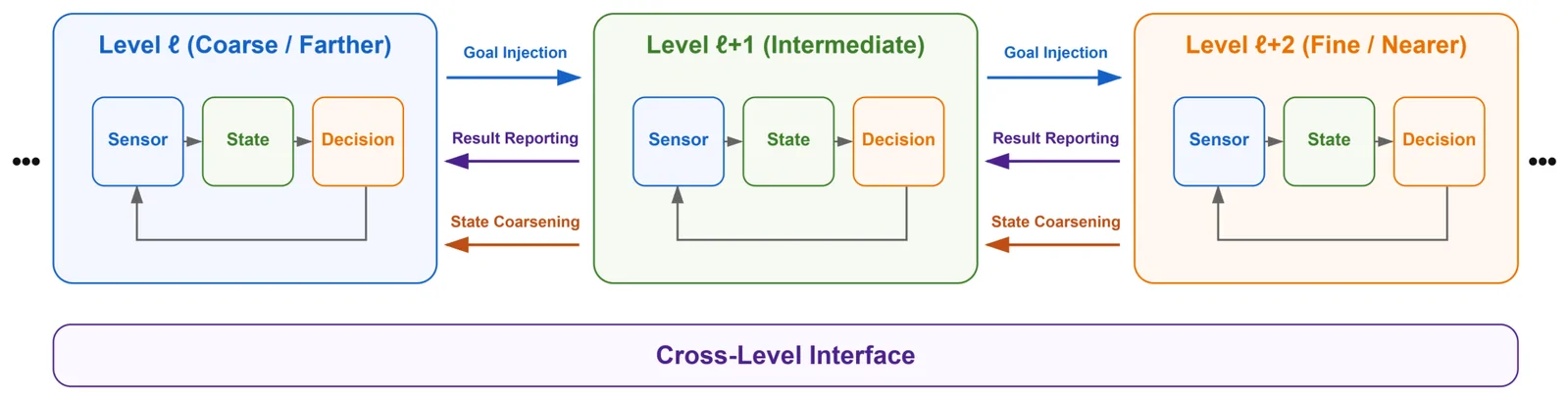
Recursive SSD hierarchy and cross-level interfaces. Each level is a complete Sensor–State–Decision loop. Adjacent levels are coupled by top-down goal injection and bottom-up result reporting and state coarsening. Coarser or farther and finer or nearer describe relative operating regimes within the hierarchy.

Figure 2 extends the single-level loop into a recursive hierarchy. Each level preserves the same Sensor–State–Decision organization. Goal injection passes tasks or constraints from a coarser parent to a finer child; result reporting returns outcomes, failures, risks, and uncertainty to the parent; and state coarsening summarizes fine-grained child states into the representation required for coarser decisions. Under this reading, driving maneuver planning and surgical contact control differ in scale and timing, but share the same cross-level interfaces.

### 4.4. Sensor Layer: The Observational Boundary

The Sensor Layer is the physical observation function of the framework. Let $S_{t}$ denote the true world state and $O_{t}=\left\{o_{t}^{\left(1\right)},\ldots ,o_{t}^{\left(K\right)}\right\}$ the observations produced by *K* sensors:

(1) $$O_{t}∼p\left(O|S_{t},\theta_{\mathrm{sensor}}\right),$$

where $\theta_{\mathrm{sensor}}$ includes noise, field of view, resolution, rate, calibration, latency, synchronization, and occlusion. Because $S_{t}$ is only partially observed [41], the sensor suite sets the upper bound on what the State and Decision Layers can use. Redundancy and cross-modal complementarity improve coverage and calibration, but also introduce synchronization and fusion uncertainty.

### 4.5. State Layer: World-State Representation

The State Layer transforms raw observations into an inferred state ${\hat{S}}_{t}$ suitable for prediction, planning, and control. Not every embedding is a world state. We use three sufficiency conditions.

#### 4.5.1. Defining World State: Three Sufficiency Conditions

1. Filtering sufficiency. ${\hat{S}}_{t}$ should summarize relevant observation history for estimating the current world:
  (2) $$p\left(S_{t}|O_{1:t}\right)\approx p\left(S_{t}|{\hat{S}}_{t}\right).$$
2. Action-conditioned predictive sufficiency. Given a candidate action $a_{t}$, the state should support prediction of future states or observations:
  (3) $$p({\hat{S}}_{t+1},O_{t+1}|{\hat{S}}_{t},a_{t}).$$
3. Decision sufficiency. The state should contain variables needed to evaluate reward, cost, risk, constraints, or value:
  (4) $$V\left(a_{t},{\hat{S}}_{t}\right)\;\mathrm{or}\;c\left(a_{t},{\hat{S}}_{t}\right).$$

Together, these conditions make world state an observation-grounded, temporally predictive, and decision-relevant representation, not a generic feature vector.

#### 4.5.2. State Composition

Embodied world states usually combine environmental state, agent state, and interaction state. Environmental state covers objects, occupancy, geometry, trajectories, and semantics. Agent state covers pose, velocity, joints, actuators, and action history. Interaction state covers contact forces, collision proximity, grasp affordance, visibility, reachability, and other relations that are neither purely environmental nor purely internal. Far-field systems emphasize layout, dynamic agents, and long-horizon scene evolution; near-field systems emphasize contact, force, compliance, and affordance.

### 4.6. Decision Layer: Prediction, Planning, and Control

The Decision Layer consumes ${\hat{S}}_{t}$ and turns it into actionable output. It supports state prediction,

(5) $${\hat{S}}_{t+1}∼p_{\phi }\left({\hat{S}}_{t+1}|{\hat{S}}_{t},a_{t}\right),$$

counterfactual rollout under candidate sequences, planning,

(6) $$a_{t:t+H}^{*}=\arg\max_{a_{t:t+H}}E\left[\sum_{h=0}^{H}\gamma^{h}r\left({\hat{S}}_{t+h},a_{t+h}\right)\right],$$

closed-loop control, and simulation-based data generation. The transition model belongs to the State Layer when it updates the world state, but becomes a decision interface when planning or control consumes its rollouts. Decision quality therefore depends on long-horizon stability, latency, uncertainty calibration, and whether the predicted variables match the task objective.

### 4.7. Uncertainty Pathway Across SSD

Uncertainty is a cross-layer interface, not only an open challenge. The core pathway is

(7) sensor uncertainty→state uncertainty→prediction uncertainty→decision risk.

At the Sensor Layer, observations should be recorded with confidence, calibration, timing, synchronization, and quality metadata. Sources include calibration error, latency, asynchronous sampling, occlusion, modality failure, adverse conditions, and distribution shift. At the State Layer, these factors affect fusion, memory, and update, appearing as occupancy probability, pose covariance, detection confidence, contact ambiguity, stochastic embeddings, ensembles, diffusion samples, or other implicit distributions. At the Decision Layer, uncertainty matters only if it changes planning, control, or safety behavior, such as risk-sensitive planning, active sensing, sensor routing, fallback policies, safety margins, or decision-sensitivity analysis.

The SSD mapping should distinguish implicit generative uncertainty from calibrated or propagated uncertainty. Where the literature does not support a strict aleatoric/epistemic split, the practical treatment should be recorded instead of overstating theory. Sensor sections should identify uncertainty sources, state sections should show how they are absorbed or exposed, architecture sections should note whether uncertainty is implicit or explicit, and evaluation sections should test whether uncertainty affects closed-loop decisions.

### 4.8. Long-Tail Propagation and Generation Across SSD

In SSD, the long tail includes not only rare categories or scenes, but low-frequency, high-consequence conditions that arise and propagate across layers. Sensor tails include adverse weather, occlusion, calibration drift, asynchronous sampling, and modality failure. State tails include rare transitions, hidden objects, contradictory evidence, or contact changes. Decision tails occur when low-probability conditions force risk-sensitive choices under uncertainty.

Long-tail scene generation is valuable because many such events are costly or unsafe to collect at scale in autonomous driving, robotic manipulation, and physical AI. However, visually plausible generation is insufficient. Generated long-tail observations should remain consistent with measurable physical states, sensor constraints, and action-conditioned dynamics; otherwise they may amplify state drift. Closed-loop recovery is the decisive test: wrong decisions can change future observations and state estimates, so the framework must evaluate whether the system detects bias, replans, and contains risk when rare failures propagate.

## 5. Sensor Layer

The Sensor Layer defines the observation boundary of a sensor-driven world model: what can be measured, at what range and resolution, how often it is updated, and with what uncertainty. We group the main modalities into exteroceptive, proprioceptive, and tactile or force sensing. Their value is not only information content, but also whether they reduce state ambiguity and decision risk.

1. Exteroceptive sensors observe the external scene. Cameras provide dense semantic evidence but weak metric depth; LiDAR supplies accurate 3D geometry but becomes sparse at range; and radar provides range and radial velocity with stronger robustness to weather and lighting, but lower spatial resolution [42,43,44].
2. Proprioceptive sensors measure the agent’s own pose, motion, joints, and executed actions through IMUs, encoders, odometry, and related channels. They support ego-motion estimation, temporal alignment, and action-conditioned transition modeling [45,46,47].
3. Tactile and force sensors measure contact forces, pressure, slip, texture, and deformation at the interaction surface. They expose contact-level variables that vision often cannot infer under occlusion, making them central to near-field manipulation, grasping, locomotion, and human–robot interaction [48].

### 5.1. Exteroceptive Sensors

Far-field world models commonly combine cameras, LiDAR, and radar because no single modality supplies dense semantics, precise geometry, and all-weather robustness at once [44,49,50]. Cameras are the dense semantic channel for lanes, signs, objects, and appearance. LiDAR is the metric backbone for localization and 3D structure, but point density decreases with distance, leaving many far objects weakly observed [51]. Radar complements both by measuring range and Doppler velocity directly and remaining useful in rain, fog, and snow [52,53,54]. Recent critical reviews of in-vehicle sensing [49] emphasize exactly this point: cameras provide rich scene information but degrade under poor lighting and weather, LiDAR delivers accurate three-dimensional geometry yet suffers signal attenuation in rain and fog, and radar offers superior robustness and direct velocity measurement but is limited in angular resolution. For SSD, fusion is therefore not an optional front-end detail: the chosen sensor suite determines which state variables can be built and how uncertainty enters planning, and the three modalities supply genuinely complementary channels—semantic, metric, and dynamic—rather than redundant ones. Table 8 summarizes the contributions, characteristic uncertainties, and SSD roles of these far-field modalities, together with GNSS/IMU sensing for ego-state alignment.

### 5.2. Proprioceptive Sensors

Proprioception reports the agent’s own state without relying on external visibility. IMUs and joint encoders operate at hundreds of hertz or more, far above typical camera and LiDAR rates, and remain available in featureless, dark, or visually degraded environments [55,56]. In world models, these signals are both state-estimation inputs and action-feedback channels. DreamerV3 shows that proprioceptive vectors alone can support world-model learning on continuous-control tasks [4], while DayDreamer fuses proprioception with images so the latent dynamics can track body pose, joint angles, and contact during real robot learning [57].

### 5.3. Tactile and Force/Torque Sensors

Tactile and force/torque sensors define the innermost near-field observation boundary. They activate at physical contact and measure forces, pressures, local geometry, slip, and deformation, which are decisive for grasping, insertion, assembly, locomotion, and surgical manipulation [48]. Vision-based optical tactile sensors such as GelSight use camera-observed elastomer deformation to recover high-resolution contact geometry, normals, and force cues [58]. Recent visuo-tactile world models show that adding fingertip tactile observations improves contact-rich imagination over vision-only prediction, especially when occlusion and multi-step physical interaction dominate [19]. Table 9 compares the complementary contributions, uncertainties, and SSD roles of visual, proprioceptive, tactile, and force/torque sensing in the near field.

The long-tail problem in sensing therefore includes not only rare external scenes such as night, rain, snow, or smoke, but also low-frequency sensor failures such as calibration drift, latency, modality dropout, occlusion, and contact-sensor saturation. Robustness at the Sensor Layer should consequently be evaluated by its effect on state stability, decision risk, and recovery, rather than by front-end accuracy alone.

## 6. State Layer: World State Representation

The State Layer receives heterogeneous observations from the Sensor Layer and converts them into an inferred representation on which the Decision Layer can predict, plan, and act:

(8) $${\hat{S}}_{t}=g_{\phi }\left(O_{1:t},a_{1:t-1}\right),$$

where $g_{\phi }$ filters observation and action history into a compact state estimate. Section 4.5.1 defines the required sufficiency conditions: the state must summarize relevant observation history, support action-conditioned prediction, and retain variables needed to evaluate candidate actions.

**Figure 3:**
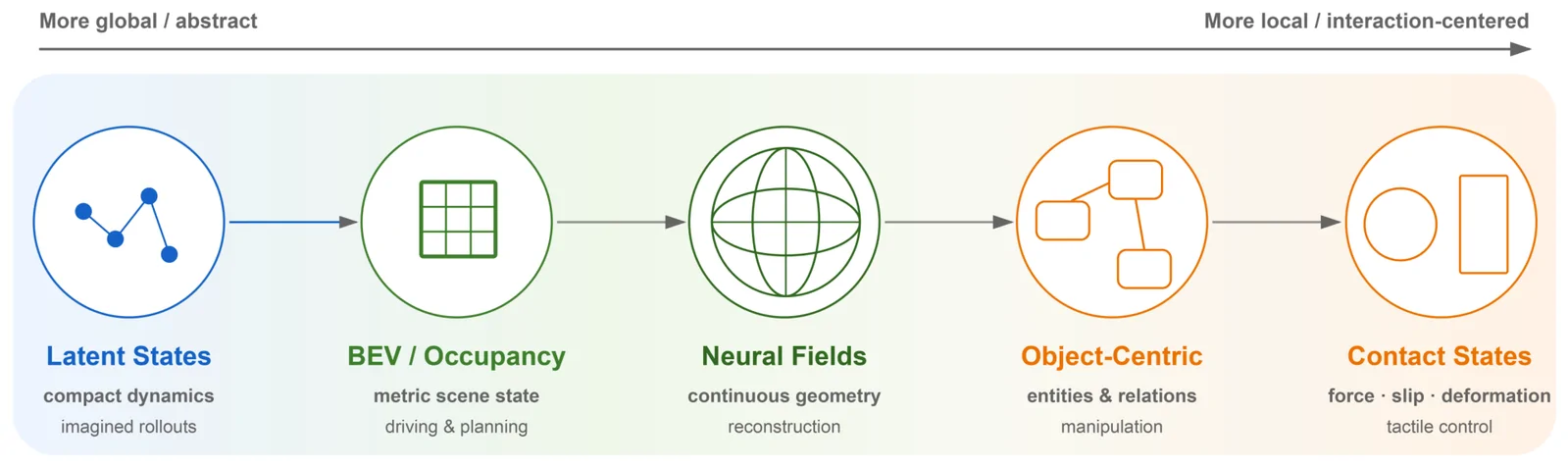
Representative state-representation families in the SSD State Layer. The landscape ranges from compact and comparatively abstract latent states toward increasingly structured, local, and interaction-centered representations. The arrangement is not a strict ordering because a world model may combine several representation forms at the same operating level.

These conditions constrain the representation without fixing its form. Current sensor-driven world models use several families of state representation, each encoding a different prior about what should be retained: compact latent dynamics, metric spatial structure, volumetric geometry, entity-level decomposition, or contact-centered interaction. Figure 3 summarizes this design space.

### 6.1. Latent Tokens

Latent-token representations compress high-dimensional observations into compact vectors and model dynamics directly in latent space. World Models [1] separated a variational encoder from recurrent dynamics; PlaNet [2] consolidated this into a recurrent state-space model; Dreamer [3] and DreamerV3 [4] optimized behavior through imagined latent trajectories; and MuZero [5] pushed the idea toward decision sufficiency by predicting reward, value, and policy rather than reconstructing observations. The strength of latent tokens is efficient rollout; the cost is weak interpretability and limited access to geometry, object relations, or calibrated uncertainty.

### 6.2. Bird’s-Eye-View Representations

Bird’s-eye-view (BEV) representations project multi-view and multi-sensor observations into a shared top-down metric frame. BEVDet [59] established a modular camera-to-BEV pipeline, BEVFormer [31] added spatiotemporal attention, BEVFusion [60] fused camera and LiDAR in the same coordinate system, and UniAD [61] carried BEV through detection, tracking, prediction, and planning. BEV is well matched to driving because it preserves layout and trajectory relations, but planar projection loses height structure and fixed grid resolution limits fine geometry.

### 6.3. Occupancy Grids and Neural Fields

Occupancy representations recover the third dimension by distinguishing occupied, free, and unobserved space. Occupancy networks [62], Occ3D [24], Scene as Occupancy [63], and OccWorld [40] show how volumetric state can support perception and future occupancy prediction. Neural rendering pursues the same volumetric goal with photometric fidelity. These states make occlusion and free space explicit, but their memory and rollout cost are high, and decision variables such as risk or affordance must often be computed on top of geometry.

### 6.4. Object-Centric Representations

Object-centric representations decompose the state into entity-level components. Slot Attention [64] learns such decompositions by assigning image regions to latent slots, and FOCUS [65] uses per-object latent dynamics for model-based robot learning. This granularity supports relational reasoning, compositional generalization, and manipulation, where object identity and affordance are decision-relevant. Its weaknesses are object discovery in clutter, fixed slot capacity, and poor fit to amorphous or deformable materials.

### 6.5. Action-Conditioned and Contact States

Action-conditioned states are trained so that their evolution under candidate actions is the primary target. Dreamer [3], DayDreamer [57], and iVideoGPT [66] illustrate this family across latent control, real robot learning, and interactive video prediction. At the closest scale, the relevant variables become contact geometry, normal and shear force, and incipient slip. Optical tactile sensing [58] makes these quantities observable, and visuo-tactile world models [19] learn their dynamics jointly with vision. Long-tail state modeling therefore requires more than rare-object recognition: under incomplete or contradictory sensing, the state must preserve multiple plausible hypotheses and risk-related variables for the Decision Layer.

## 7. Decision Layer: Prediction, Planning, and Control

The Decision Layer turns world-state representations into executable behavior or explicit decision support. It uses the state produced by the Sensor and State Layers to predict future states, compare alternatives, plan actions, and, where a controller is present, close the feedback loop. Its quality is therefore measured not only by prediction accuracy, but also by whether predictions remain useful under latency, uncertainty, and changing task conditions.

### 7.1. World State Prediction

Prediction is the base capability for the rest of the layer:

(9) $${\hat{S}}_{t+1:t+H}=f_{\theta }\left({\hat{S}}_{t},a_{t:t+H-1}\right),$$

where ${\hat{S}}_{t}$ is the current state estimate and $a_{t:t+H-1}$ is a candidate action sequence. World Models [1], PlaNet [2], Dreamer [3], and DreamerV3 [4] show how latent dynamics can support policy learning through imagined trajectories. Visual and driving systems extend the same idea to action-conditioned video, occupancy, and controllable scene generation, as in iVideoGPT [66], OccWorld [40], and DriveDreamer [21]. The main difficulty is long-horizon stability: small model errors can accumulate into states that remain visually plausible but no longer preserve decision-relevant geometry, contact, or risk.

### 7.2. Counterfactual Reasoning

Counterfactual reasoning compares futures under different actions before any one of them is executed. Dreamer and DreamerV3 [3,4] implement this through latent imagination, while iVideoGPT [66] extends action-conditioned comparison to video prediction. This function distinguishes world-model-based agents from reactive policies: the agent can ask what would follow from braking, turning, grasping, pushing, or waiting, and can choose according to expected task value and risk.

### 7.3. Planning

Planning searches the learned model for an action sequence worth executing:

(10) $$a_{t:t+H}^{*}=\arg\max_{a_{t:t+H}}E\left[\sum_{h=0}^{H}\gamma^{h}r\left({\hat{S}}_{t+h},a_{t+h}\right)\right].$$

PlaNet [2] is the canonical latent-planning example, and the Dreamer family folds this search into actor-critic learning. In driving, UniAD [61] connects perception, prediction, and planning around the final planning objective. Compared with model-free control, planning through a world model can improve sample efficiency and generalization, but only if the state exposes the variables that the planner must optimize or avoid.

### 7.4. Control

Control executes planned actions and corrects them with new observations. DayDreamer [57] demonstrates this loop on real robots, while Pronto [55] illustrates why high-rate inertial and proprioceptive sensing matters for stable locomotion. Driving usually operates at trajectory-level frequency and meter-scale precision; manipulation and surgical robotics require contact-level feedback at much higher rates. The Decision Layer is therefore constrained by latency, sensor update rate, rollout cost, and the precision of the state variables it receives.

### 7.5. Simulation and Data Generation

A world model can also serve as a simulator for training, testing, or data augmentation. DreamerV3 [4] learns policies from imagined rollouts, and DriveDreamer [21] synthesizes controllable driving futures. This is especially useful for rare, unsafe, or expensive scenarios, but generated experience is valuable only when the simulated dynamics preserve the decision-relevant structure of the real environment. From a long-tail perspective, the central question is whether the model can detect low-probability, high-consequence deviations and replan safely when new observations contradict the imagined future.

## 8. Sensor-Driven World Model Architectures

Rather than classify world models by learning paradigm, this section organizes architectures by sensor composition and information flow.

Table 10 compares the main families by observations, state representation, decision target, evaluation setting, SSD regime, and key limitation. It complements the system-level mapping in Table 5, which codes individual systems.

### 8.1. Vision-Centric Architectures

Vision-centric architectures infer environment dynamics from images or video. Their canonical pipeline encodes observations into a latent state, advances that state with a dynamics model, and supports planning or policy learning. World Models [1] established latent-simulator control, PlaNet [2] coupled recurrent latent dynamics with online planning, Dreamer [3] learned policies from imagined trajectories, and DreamerV3 [4] stabilized the approach across diverse tasks.

A generative branch replaces compact recurrent states with internal video-model representations. GAIA-1 [20] and DriveDreamer [21] generate controllable driving futures, Vista [67] emphasizes fidelity and cross-scenario generalization, and Genie [9] infers latent actions from unlabeled video to create controllable environments. These systems gain visual fidelity and data scale at the cost of more expensive rollouts. The two branches therefore emphasize different state abstractions: latent-control models favor compact imagined rollouts and decision efficiency, whereas generative video models favor observable fidelity and broader training distributions. Beyond autonomous driving and games, the same generative line extends to bandwidth-efficient media production, where emotion-aware scene adaptation [68] uses semantic and reinforcement signals to generate animated content that preserves the affective content of source video; this same trade-off between compactness and observable fidelity recurs in those content-creation workflows.

Vision-centric models offer broad semantic coverage and excellent data scalability, but monocular pixels do not directly provide metric geometry or contact state. Both branches remain bounded by visual observability and are strongest in macroscopic settings where scene semantics matter more than fine physical interaction.

**Table 10:** Landscape comparison of representative sensor-driven world-model architecture families.

Architecture Family	Representative Systems	Sensor Modalities	State Representation	Prediction/Decision Target	Representative Data or Evaluation Setting	Typical Metrics or Criteria	SSD Regime	Key Limitation
Vision-centric	World Models [1], PlaNet [2], DreamerV3 [4], GAIA-1 [20], DriveDreamer [21], Genie [9]	Image or video observations	Compact latent dynamics or generative video latent states	Future latent or visual observations; policy learning; scenario generation	Simulated control tasks, driving video, and large-scale video corpora	Task return or success; future-prediction fidelity; controllability	Mostly macroscopic or abstract single-level SSD	Visual scale and semantic coverage are strong, but metric geometry and contact variables are not directly observed.
Camera–LiDAR–radar fusion	BEVFusion [60], FusionRCNN [69], CenterFusion [54], TransFusion [70], UniAD [61]	Camera, LiDAR, radar, ego-motion sensors	BEV, occupancy, fused object or trajectory states	3D perception, motion prediction, occupancy, and trajectory planning	nuScenes [50], K-Radar [53], and driving planning benchmarks	mAP, NDS, IoU, trajectory error, and planning or collision criteria	Far-field/macroscopic SSD	Fusion improves observability but adds calibration, synchronization, computation, and modality-degradation dependencies.
Vision–proprioception–action	DayDreamer [57], DreamerV3 [4], RoboDreamer [71]	Vision, joint or inertial state, proprioception, executed actions	Action-conditioned latent state with embodied state variables	State transition, skill prediction, policy learning, and closed-loop control	Physical robot interaction and manipulation tasks	Task return, success rate, control stability, and prediction consistency	Mesoscopic/embodied-control SSD	Embodiment information improves control fidelity, but the representation remains tied to platform-specific body states and has limited global spatial reach.
Vision–tactile/force	Visuo-Tactile World Models [19] and GelSight-class sensing pipelines [58]	Vision or RGB-D, tactile sensing, force/torque sensing	Contact-state and interaction-centered latent representations	Contact evolution, slip, grasp stability, force-aware manipulation	VisGel [72], TaF-Dataset [73], and contact-rich manipulation tasks	Contact-state prediction, force estimation, grasp or manipulation success	Near-field/contact-level SSD	The observable region is local and informative contact events are expensive, brief, and often hardware-specific.
Hybrid and hierarchical	Director [74] and multi-level sensor-state architectures reviewed in Section 8.5	Level-specific combinations of exteroception, proprioception, and contact sensing	Multiple states at different spatial, temporal, or task granularities	Sub-goal generation, local execution, result reporting, and cross-level replanning	Hierarchical control tasks; the complete cross-scale sensor hierarchy remains sparsely instantiated	Task return and goal completion; cross-level recovery and consistency remain underdeveloped	Recursive far-field/near-field SSD	Individual levels are well represented in the literature, but explicit goal injection, result feedback, and state coarsening across sensor-distinct levels remain rare.

### 8.2. Camera–LiDAR–Radar Fusion Architectures

Camera-only driving perception lacks reliable metric geometry and becomes vulnerable when optical sensing degrades. Fusion architectures add complementary range sensors, with the main design choice being where the modalities interact.

Early fusion combines raw or feature-level signals, mid-level fusion aligns modalities in a shared representation such as BEV, and late fusion reconciles modality-specific outputs. The choices impose different trade-offs: early fusion preserves low-level complementarity but requires tight alignment, shared spatial fusion offers a common geometric state but remains calibration-sensitive, and late fusion is modular but delays cross-modal correction. BEVFusion [60] exemplifies shared-BEV fusion; FusionRCNN [69] and CenterFusion [54] refine detections with image, LiDAR, or radar cues; TransFusion [70] uses attention to improve tolerance to sensor degradation and calibration error; and UniAD [61] carries the fused state through detection, tracking, prediction, and planning so that representation learning is aligned with the planning objective.

4D radar further preserves range and velocity measurements in rain, fog, and snow, and K-Radar [53] has helped make radar-centric fusion a practical direction. These architectures improve geometry and adverse-condition robustness, but they also increase calibration, synchronization, computation, and modality-degradation dependencies. End-to-end stacks such as UniAD reduce separation between perception and planning, while making failures and optimization more tightly coupled across modules.

### 8.3. Vision–Proprioception–Action Architectures

Visual observations alone can map similar frames to different body configurations and velocities. These architectures therefore add joint, inertial, proprioceptive, and executed-action signals so that state transitions are conditioned on the agent’s own embodiment.

DayDreamer [57] demonstrates direct world-model learning on physical robots, and DreamerV3 [4] uses the same closed sensorimotor principle. In legged robotics, fused kinematics and inertial sensing provide control-rate state estimates that vision alone cannot supply [56]; in manipulation, RoboDreamer [71] predicts action-conditioned futures for robot arms. This modality mix is especially valuable when rapid body motion or contact makes external observations delayed or ambiguous. Proprioception improves control fidelity by closing the sensorimotor loop, although the resulting state remains tied to platform-specific embodiment, which limits transfer across morphologies and offers less global spatial reach than driving-oriented representations.

### 8.4. Vision–Tactile/Force Architectures

Near contact, occlusion weakens vision and variables such as pressure, slip, and force are not directly observable. Visuo-tactile architectures therefore combine external vision with contact sensing. GelSight-class devices [58] convert contact into high-resolution images, broader tactile work expands the available hardware and data [48], and visuo-tactile world models [19] predict action-conditioned contact dynamics from fused observations. Unlike long passive video streams, tactile evidence is sparse and concentrated in short interaction intervals. These states support manipulation under occlusion, but local sensing, hardware specificity, and scarce contact events limit scale and transfer.

### 8.5. Hybrid and Hierarchical Architectures

Figure 2 defines the cross-level interfaces, while Table 11 arranges reviewed architectures as complete SSD loops from coarse to fine. Levels follow the multidimensional operating profile defined in Section 4, rather than fixed sensor suites or distance bands; $\lambda_{\ell }$ and $\tau_{\ell }$ remain illustrative descriptors of spatial extent and decision timescale. Existing work populates scene-level, object- or skill-level, and contact-level loops, but most systems remain within one level. The recursive reading does not imply that every system requires several levels; it identifies when multiple operating scales are present and whether their interfaces are explicit. The principal gap is cross-level coupling through goal injection, result feedback, and state coarsening.

Director [74] is the clearest hierarchical world-model example in the reviewed literature. Built on PlaNet and Dreamer [2,3], it learns a manager that selects goals and a worker that pursues them using a shared latent state from one world model over image and proprioceptive inputs. Goal injection is only partial: the manager outputs a point in the shared latent space, discretized by a goal autoencoder, and the worker is rewarded for matching that point rather than operating as a child model with its own state, objective, and sufficiency conditions. The hierarchy is therefore mainly a decision hierarchy over one shared representation, rather than a hierarchy whose levels maintain distinct sensor profiles and state abstractions. Director demonstrates hierarchical control without realizing a fully sensor-distinct recursive SSD hierarchy.

#### Architecture-Level Synthesis

Across the families, range sensing, proprioception, and contact sensing progressively expose geometry, embodiment, and interaction variables that vision alone cannot observe. The architecture families therefore differ most fundamentally in what parts of the world become observable and decision-relevant, rather than only in learning objective or backbone. This progression is not a ranking: the appropriate architecture depends on the state variables required by the downstream decision. The main structural gap lies between levels, because single-level solutions are comparatively mature while explicit goal injection, result feedback, and state coarsening remain sparse.

## 9. Far-Field Perception and Near-Field Observation

Far-field and near-field sensing provide complementary views of embodied environments. Far-field sensing typically captures broad scene context, dynamic agents, and longer-range motion using cameras, LiDAR, radar, and positioning sensors, whereas near-field sensing resolves local geometry, contact, force, deformation, and fine interaction through depth, tactile, force/torque, joint, and proprioceptive measurements. A unified world model must maintain temporally consistent states across these heterogeneous observations so that both global planning and local interaction remain decision-relevant.

**Figure 4:**
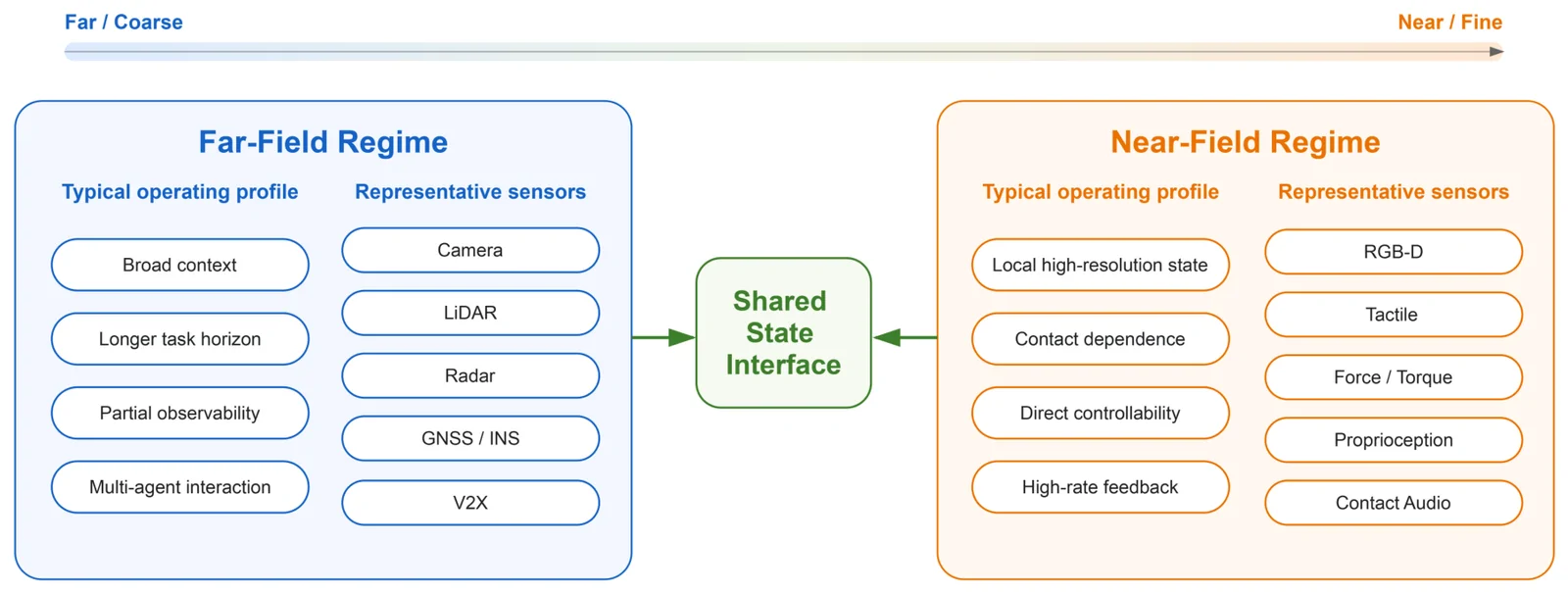
Relative far-field and near-field operating regimes in the recursive SSD hierarchy. Far-field operation typically emphasizes broad context, longer horizons, partial observability, and multi-agent interaction, whereas near-field operation emphasizes local high-resolution state, contact dependence, direct controllability, and high-rate feedback. The representative sensor sets illustrate common modality choices but do not define the regimes.

Figure 4 treats far-field and near-field as relative operating regimes, not geometric bins. Sensing range remains informative, but the classification jointly depends on context, spatial and temporal resolution, uncertainty, task horizon, controllability, contact state, and control frequency. Table 8 and Table 9 provide the modality-level view, and the driving and surgical cases apply the same criteria in contrasting domains.

### 9.1. Unified Far-Field and Near-Field

A unified representation must connect broad environmental structure with fine physical interaction. Cameras, LiDAR, and radar support road geometry, dynamic agents, traversable regions, and large-scale spatial relations, while depth, tactile, and force sensing reveal local geometry, contact state, deformation, and force. BEV representations provide a common spatial basis for heterogeneous far-field observations, and tactile sensing supplies contact information unavailable to vision [60]. The resulting world state should preserve both global context and local interaction variables, with explicit relations that can be queried and updated as the task shifts between scales.

#### 9.1.1. Hierarchical and Multi-Resolution Scene Reconstruction

Hierarchical reconstruction can encode large environments as BEV maps, 3D occupancy fields, semantic maps, or object-centric graphs while reserving dense point clouds, implicit surfaces, contact maps, and mechanical states for action-relevant regions. NeuralRecon incrementally fuses temporally coherent local geometry, and Vox-Fusion expands voxel-based neural implicit maps as the explored area grows [75,76]. The main advantage is selective resolution: the model can retain fine spatial and temporal detail around objects that affect the current decision while compressing distant or stable regions into coarser states. Such allocation also provides a natural state-coarsening interface between adjacent SSD levels.

#### 9.1.2. Cross-Modal and Cross-Scale Consistency

Cross-scale states should remain consistent without suppressing uncertainty or genuine sensor disagreement. Far-field object estimates from cameras and LiDAR should remain compatible with near-field depth, tactile, or force evidence when the observations refer to the same interaction. TransFusion shows that soft cross-modal association can reduce sensitivity to degraded images and calibration errors [70]. However, consistency does not require forced agreement. Tactile evidence may reveal a slip before a visual change is apparent, and asynchronous sensors may temporarily support different hypotheses. The model should preserve such discrepancies, propagate their uncertainty, and raise local risk when the disagreement affects a decision.

#### 9.1.3. Physically Consistent Synthetic Simulation

The same state interface can connect reconstructed scenes with synthetic simulation. Far-field simulation must preserve layout, traffic participants, sensing conditions, and longer-horizon motion, whereas near-field simulation must capture collision, friction, deformation, force, and tactile response. UniSim converts recorded multimodal driving logs into editable closed-loop sensor simulations, while TACTO provides configurable high-resolution vision-based tactile simulation [77,78]. The relevant criterion is therefore not visual realism alone. Synthetic observations should remain geometrically and temporally coherent across modalities and should respond physically plausibly when the agent changes its action.

#### 9.1.4. Bidirectional Calibration Between Reality and Simulation

Reality and simulation should also calibrate each other. Real observations can estimate geometry, dynamics, friction, sensor noise, latency, and other parameters that determine simulator fidelity, while simulation can expose rare or unsafe transitions that are difficult to collect physically. Domain randomization improves transfer through broad simulated variation, and simulator calibration uses real trajectories to fit parameter distributions that better reproduce observed behavior [79,80]. The loop separates two complementary roles: real data anchor common physical behavior, while simulation expands coverage of low-frequency conditions. New physical evidence can then correct systematic simulation bias.

#### 9.1.5. Action-Conditioned Behavior Generation

A decision-oriented world state must support action-conditioned prediction, not only reconstruction. The model should estimate how candidate actions change the future states of the agent, surrounding objects, and other participants, which enables counterfactual comparison before execution. GameFormer jointly reasons about ego planning and surrounding traffic behavior. The same principle can compare braking, lane changes, grasps, pushes, or insertions according to expected safety, task value, uncertainty, and recoverability. A visually accurate prediction is insufficient if the state does not preserve variables needed to distinguish these action consequences.

#### 9.1.6. Hierarchical Decision Making and Closed-Loop Control

Decision making should preserve the same hierarchy. High-level policies select routes, goals, skills, or task stages; intermediate modules form trajectories or interaction plans; and low-level controllers correct execution from visual, tactile, and force feedback. UniAD links perception, prediction, mapping, and planning around a planning objective, while SayCan constrains high-level task proposals by executable robot skills [61]. Far-field states therefore support strategic anticipation, whereas near-field states support rapid correction during execution. The hierarchy also requires a reverse path: contact failure, local infeasibility, or rising uncertainty must be able to trigger changes in trajectories, goals, or task sequences at the parent level.

#### 9.1.7. Decision-Oriented Evaluation of Unified World Models

Evaluation should test decision quality rather than reconstruction or image fidelity alone. Relevant criteria include the preservation of decision-critical information across abstraction levels, physically plausible responses to actions, risk over multiple closed-loop steps, and recovery from rare disturbances. nuPlan demonstrates planning-specific closed-loop evaluation for driving, while ManiSkill2 provides interactive protocols across manipulation tasks, observations, and controllers [81,82]. These benchmarks also expose a broader requirement for unified world models: evaluation should connect perception and prediction metrics to planning, control, uncertainty calibration, and long-tail recovery rather than treating each component independently.

**Figure 5:**
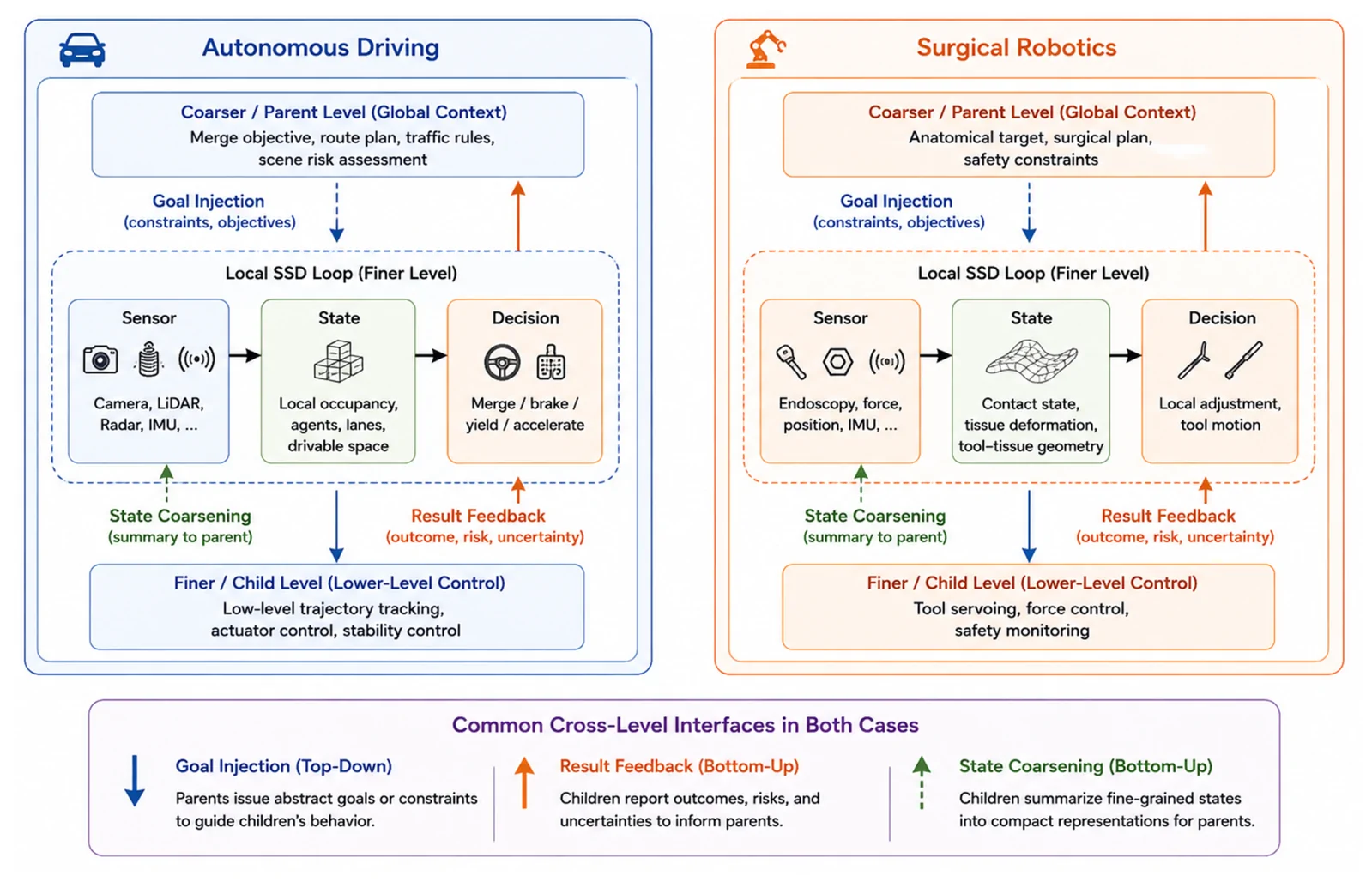
Worked examples of recursive SSD operation in autonomous driving and surgical robotics. Both domains follow the same five-step pattern: goal injection, sensor evidence, state construction and coarsening, decision or action, and result feedback. The domain-specific differences lie in the operating regime and the state variables required for safe decisions.

Figure 5 applies the recursive SSD interfaces to two contrasting domains. The driving trace emphasizes scene context, occlusion, and maneuver-level replanning, while the surgical trace emphasizes contact state, high-rate correction, and safety-critical escalation. Section 9.2 and Section 9.3 examine the two cases.

### 9.2. Autonomous Driving Applications

Autonomous driving represents a coarser operating regime because route and maneuver decisions require broad context, multi-agent interaction, partial observability, longer horizons, and trajectory-level updates. Observations often span tens or hundreds of meters, but that extent is descriptive rather than definitional. The hierarchy is also temporally nested: a maneuver such as overtaking or merging contains a trajectory process, and the trajectory contains faster lower-level control loops.

A representative long-tail case combines occlusion with adverse weather: a large vehicle hides an adjacent lane until another road user cuts into view. The difficulty is not only detecting the object after it appears. An SSD world model must preserve plausible hypotheses about the hidden region, update them as new sensor evidence arrives, and expose residual uncertainty to the decision layer. DriveDreamer-2 generates specified rare events such as cut-ins, while Vista targets generalization across unseen driving environments [22,67]. The scenario therefore tests both long-tail generation and uncertainty-aware closed-loop response.

Figure 5 traces one recursive SSD cycle. A maneuver-level parent injects the merge goal and safety constraints. Cameras, LiDAR, radar, and ego-state sensing provide partial evidence, while the finer loop updates agent and occupancy hypotheses. State coarsening returns collision risk, feasible gap, and residual uncertainty rather than the full local state. The decision interface then compares merge, brake, and yield actions. If the hidden vehicle appears or uncertainty exceeds the local safe range, result feedback triggers maneuver-level replanning. The levels therefore differ by context, horizon, uncertainty, and decision granularity rather than a fixed distance threshold.

Early driving world models depended heavily on structured conditions such as HD maps or recorded video, which constrained scalable generation of rare scenarios. Panacea and Panacea+ use cross-view and cross-temporal multi-camera modeling for panoramic synthesis [83,84]; MagicDrive and MagicDrive-V2 preserve sensor geometry in higher-resolution and longer video generation [85,86]; and WoVoGen projects multiple sensor views from a shared 4D world representation [87]. Drive-WM and ReSim further connect visual prediction with action-conditioned decision processes [88,89], while DriveDreamer-2 supports language-specified rare maneuvers and hazardous events [22].

Despite this progress, much of the literature remains camera-centric. Sensor-driven analysis must also account for asynchronous fusion, modality loss, calibration drift, and degradation propagation across camera, LiDAR, radar, and GNSS/IMU streams. These failures matter because a state can remain visually plausible while its metric geometry, velocity estimate, or localization becomes unreliable. Robust world models therefore need to represent sensor provenance and uncertainty through the state and decision interfaces, not only at the perception front end.

### 9.3. Surgical Robotics Applications

Surgical robotics represents a finer, contact-dominated regime for suturing, cutting, puncture, and endoscopic soft-tissue manipulation. Its near-field character follows from local high-resolution state, short decision timescales, direct controllability, contact dependence, and the need to supplement vision with tactile or force feedback. The confined workspace is typical but not definitional. Small state or force errors can have immediate consequences, so rapid correction and escalation are central requirements.

The SSD reading is consistent with established surgical autonomy formulations that separate perception-to-state estimation from cost-guided state-to-action policies [90].

Endoscopic and microscopic cameras provide tissue appearance and instrument position, while tactile and force sensors reveal pressure, contact geometry, stiffness, and other variables that become decision-critical after contact. A surgical state must therefore represent hidden physical quantities such as deformation, tension, and resistance to stretching or cutting, which makes contact-aware representations essential. The contrast with driving is structural: broad metric scene models dominate before contact, whereas surgical decisions can depend on local physical variables that have no direct visual signature and change at much higher feedback rates.

The surgical trace in Figure 5 follows the same interfaces at a smaller physical scale. Anatomical navigation injects an instrument target and safety constraints. Endoscopic vision and proprioception support positioning, while tactile and force sensing become dominant at contact. The finer loop maintains contact geometry, force, slip, and local tissue response; state coarsening returns contact stability and constraint satisfaction rather than the high-rate sensor stream. Local control adjusts tool motion, and unexpected slip, deformation, or force excursions return result feedback that can trigger replanning of the instrument approach or anatomical route. The near-field level is therefore defined by interaction and control requirements even within a sub-meter workspace.

Existing medical world models illustrate two distinct uses. MeWM uses pre-treatment CT, tumor segmentation, and treatment information to form an image-state-decision chain for candidate treatment support [91]. Cosmos-H-Surgical instead treats the world model as a data engine: it learns from surgical video, generates paired video-action data, and uses those pairs to train a surgical vision–language–action policy [92]. The two uses therefore rely on different sensor and supervision chains.

In surgical robotics, long-tail risk often appears as coupled Sensor–State–Decision failure: camera-pose changes degrade visual evidence, while tissue slip or abnormal deformation corrupts interaction state, and the resulting errors can amplify each other before a high-consequence decision. Rare events are therefore not limited to unusual images. They also include uncommon contact transitions, force excursions, and combinations of sensing errors that invalidate the state used by the controller.

### 9.4. Interpreting the Far-Field/Near-Field Hierarchy

The surgical stack makes the relational far-field/near-field distinction especially clear. Anatomical navigation maintains landmarks and organ boundaries and issues positioning goals; instrument positioning combines endoscopic vision with proprioception and issues contact goals; and tissue-interaction control maintains contact state and executes motion under inherited safety constraints. The complete hierarchy can occupy less than a cubic meter, so absolute distance cannot separate its levels. Their operating profiles instead differ in spatial and temporal resolution, task horizon, controllability, contact dependence, uncertainty, and control frequency. Moreover, slip or unexpected deformation is result feedback that must propagate upward when safe recovery requires replanning the instrument approach or anatomical route. Cross-level escalation is therefore a patient-safety requirement, not merely an efficiency mechanism.

### 9.5. Summary

Autonomous driving exemplifies a coarser, context-dominated regime with multi-agent interaction, partial observability, and longer planning horizons; surgical robotics exemplifies a finer, contact-dominated regime with local physical state, direct controllability, short timescales, and high-rate tactile or force feedback. Distance differs greatly between the domains but does not define the regimes. Both cases preserve the same recursive SSD interfaces: goal injection, state coarsening, and result feedback between adjacent levels.

## 10. Datasets and Benchmarks

World-model benchmarks should ideally provide four capabilities: synchronized multimodal observations, temporally dense sequences that capture state transitions and action effects, annotations that link observations to actions, and closed-loop protocols in which actions alter subsequent observations. Few existing datasets satisfy all four simultaneously.

Dataset value in SSD depends on the research question. Table 12 compares representative resources by sensor and temporal structure, state and action annotations, operating regime, long-tail coverage, and closed-loop support. It complements the domain-specific tables by showing which SSD interfaces each resource can evaluate.

The matrix exposes structural trade-offs. Driving resources offer synchronized exteroception and geometric annotation but are usually offline; manipulation resources contain richer action and embodiment data but vary across platforms; tactile and surgical corpora expose near-field contact variables but remain smaller and hardware-specific. Simulators enable closed-loop intervention, whereas recorded data better preserve physical realism. Dataset choice should therefore follow the target SSD interface rather than scale alone. The same dataset may be adequate for representation learning yet insufficient for evaluating action-conditioned prediction, recovery, or decision safety across the complete embodied control loop.

### 10.1. Autonomous Driving Datasets

Driving datasets place strong demands on synchronization because cameras, LiDAR, radar, GPS, and IMU must align while supporting forecasting and safe trajectory reasoning. Table 13 summarizes representative resources. KITTI established multimodal evaluation for detection, odometry, flow, and tracking, but its short sequences and sparse interaction labels limit dynamics learning. nuScenes adds panoramic multimodal coverage and temporal continuity, while the Waymo Open Dataset extends scale and annotation density for long-horizon prediction.

Two developments move these resources closer to world-model evaluation. Occupancy approaches such as Scene as Occupancy and OccWorld [40,63] represent dense geometry and free space rather than sparse boxes, which better matches the state consumed by collision reasoning and planning. K-Radar adds synchronized 4D radar for adverse conditions in which optical sensing degrades. CARLA [99] supplies controllable traffic, weather, and interaction, allowing actions and counterfactual conditions to change subsequent observations. The distinction is important because recorded driving logs provide stronger sensor realism but cannot directly test how prediction errors propagate after an alternative action is executed. These resources occupy complementary SSD roles. KITTI, nuScenes, and Waymo primarily support Sensor-to-State questions in geometry, fusion, tracking, and forecasting; Occ3D emphasizes explicit occupancy state; nuPlan reaches the State-to-Decision interface through closed-loop planning evaluation; K-Radar tests sensor reliability; and CARLA enables intervention that recorded logs cannot provide. The comparison also separates scale from capability: a larger offline corpus may improve representation learning without providing evidence that the resulting state supports safe closed-loop decisions. No single benchmark combines sensing realism, long-tail density, rich state annotation, and closed-loop intervention for complete SSD evaluation.

**Table 13:** Representative datasets for autonomous driving world-model research.

Dataset	Main Data and Annotations	Representative Research Use Cases
KITTI [100]	Stereo RGB, LiDAR, GPS/IMU, 3D boxes, tracking, flow, and odometry.	3D detection, tracking, depth, scene flow, and visual odometry.
nuScenes [50]	Surround cameras, LiDAR, radar, GPS/IMU, HD maps, boxes, and trajectories.	Sensor fusion, BEV perception, tracking, occupancy, and forecasting.
Waymo Open Dataset [101]	Multi-camera and LiDAR sequences with boxes, tracks, maps, and trajectories.	Large-scale detection, tracking, motion forecasting, and scene modeling.
Argoverse 2 [102]	Camera and LiDAR sequences with HD maps, tracks, trajectories, and map changes.	Motion forecasting, map reasoning, tracking, and cross-city generalization.
nuPlan [81]	Driving logs with camera, LiDAR, vehicle states, maps, tracks, and plans.	Planning, imitation learning, trajectory generation, and closed-loop evaluation.
Occ3D [24]	Multi-view camera and LiDAR data with semantic occupancy and visibility labels.	Occupancy prediction, 3D reconstruction, free-space reasoning, and collision analysis.
K-Radar [53]	4D radar, LiDAR, cameras, GPS/IMU, and 3D boxes under varied weather.	Radar detection, all-weather perception, sensor fusion, and state estimation.

### 10.2. Robot Manipulation Datasets

Manipulation datasets prioritize interaction annotation because the target is an action-conditioned transition rather than scene perception alone. RLBench [93] pairs RGB-D observations, robot state, language, and actions in an interactive task suite, as summarized with other resources in Table 14. Open X-Embodiment emphasizes heterogeneity across robots and supports transfer across embodiments, whereas DROID emphasizes real household scenes, diverse object interactions, and teleoperated trajectories. The latter is valuable because contact dynamics and execution errors are difficult to reproduce faithfully in simulation. Together with real-robot results such as DayDreamer, these resources shift world modeling toward proprioceptive, action-conditioned dynamics rather than visual prediction alone. Their SSD roles remain complementary. RLBench supports controlled transition and planning studies, Open X-Embodiment supports cross-embodiment transfer, DROID tests real-world diversity, and RH20T adds force and audio for contact-aware state construction. Cross-platform aggregation increases coverage but also mixes sensor calibration, control conventions, and embodiment-specific state variables, which complicates a unified State representation. Contact-rich datasets provide greater physical detail, but usually across fewer embodiments and environments. The relevant trade-off is therefore the breadth of transfer against consistency and physical resolution of the state-action interface.

### 10.3. Tactile and Force Datasets

Tactile and force datasets remain limited in the temporal density and interaction annotation required by near-field world models. GelSight [58] enables high-resolution observation of contact geometry, force, and slip, but collection remains expensive and hardware-specific. Differences in sensing principles, calibration, and resolution also hinder transfer. Many corpora record isolated contact snapshots, although near-field control requires continuous sequences that connect action, contact transition, force evolution, and outcome at high temporal resolution. Visuo-tactile world modeling [19] begins to address this gap by predicting contact evolution from fused vision and touch, including periods in which visual evidence is degraded by manipulation-induced occlusion. For SSD analysis, VisGel, Touch and Go, and TVL mainly support cross-modal representation learning; TaF-Dataset aligns tactile and force signals for contact-state construction; and Feeling of Success links contact evidence to task outcome. These resources therefore cover different portions of the Sensor-to-State-to-Decision path rather than forming interchangeable tactile benchmarks. Most remain recorded corpora rather than closed-loop environments in which a predicted contact state changes the next action and observation, leaving action-conditioned recovery and contact-level replanning weakly evaluated.

### 10.4. Surgical and Medical Multimodal Datasets

JIGSAWS and the Cholec datasets mainly support procedure state, workflow, and action understanding from video and kinematics, while SurgSync combines synchronized vision, kinematics, and contact sensing for near-field tool-tissue states. HCC-TACE-Seg extends the scope to longitudinal clinical state and decision support but lacks an embodied sensor-action loop. The distinction matters because procedure recognition, contact-state prediction, and clinical outcome modeling correspond to different SSD interfaces and timescales. Modalities, executable actions, and outcomes therefore remain fragmented across datasets rather than being observed in one continuous embodied process. The current surgical landscape still lacks a benchmark that jointly covers multimodal observation, contact-state evolution, executable intervention, and closed-loop outcome evaluation across the complete SSD chain. Such a benchmark would need synchronized sensing at both procedural and contact scales, explicit action traces, and outcome signals that permit local control failures to be related to higher-level surgical decisions.

### 10.5. The Long-Tail Dataset for World Models

Long-tail resources fall into two categories: direct tail datasets that collect rare events and adverse sensing conditions, and diversity-oriented datasets that broaden tasks, scenes, objects, platforms, or sensors. Driving resources often target rare objects, anomalies, severe weather, sensor degradation, or accident evolution, whereas manipulation benchmarks more often test generalization beyond common task and contact combinations. The distinction separates explicit observation of low-probability failures from broader distributional coverage that may only reduce the chance of encountering an unseen combination. CODA, Fishyscapes, LostAndFound, DoTA, and related driving datasets directly expose tail-specific Sensor and State failures. Open X-Embodiment, DROID, and BridgeData V2 instead broaden the training distribution across tasks, scenes, objects, and embodiments. Direct tail sets can measure performance under named failure conditions, while diversity-oriented resources mainly test whether learned representations and policies transfer beyond frequent training combinations. SSD evaluation should report the two forms of coverage separately because they test different failure modes and support different safety claims.

### 10.6. Evaluation Metrics

Accuracy-centered protocols do not fully measure world-model value for planning, control, and safety. Five gaps are especially important:

1. Metrics such as mAP, IoU, NDS, ADE, and FDE measure perception or prediction accuracy but do not guarantee safe decisions.
2. Open-loop comparisons cannot expose error accumulation after predictions affect actions and future observations.
3. Heterogeneous sensor combinations lack unified cross-modal criteria for fair comparison.
4. Far-field and near-field benchmarks evaluate different states and decisions, although deployed embodied systems must coordinate both regimes.
5. A single composite score can hide which layer of a hierarchical SSD system causes failure.

The evaluation protocol should therefore report layer-specific errors together with downstream decision consequences. For hierarchical SSD systems, useful measures should distinguish sensing degradation, state-estimation error, prediction uncertainty, planning risk, and recovery after feedback, rather than compressing them into one accuracy score. Closed-loop evaluation is especially important because small Sensor or State errors may remain harmless in open-loop prediction yet become consequential after repeated action and feedback cycles.

### 10.7. Summary

Datasets have progressed from static single-modal perception toward multimodal temporal observation, interaction, and embodied decision making, while evaluation remains dominated by perception and prediction accuracy. Future benchmarks should combine richer multimodal sensing with action traces, uncertainty labels, and intervention protocols, especially for long-tail events. The objective should extend beyond mean error to whether decision-critical state information survives abstraction and supports safe recovery. World models should represent multiple plausible states with calibrated confidence and risk, and benchmarks should extend from single-step open-loop errors to multi-step closed-loop evaluation of risk exposure, decision stability, recovery, and worst-case performance.

## 11. Open Technology Issues and Potential Directions

Although sensor-driven world models have shown strong potential in embodied intelligence, their development is still constrained by several unresolved problems. As mentioned earlier, existing world models still model “ideal sensors” and “ideal world environments,” while the real world often needs to meet long-tail deployment requirements, namely “asynchronous deployment, fragmentation, diverse interpretations, and uneven risk.” Therefore, the possible solutions not only include improving prediction accuracy but also incorporating spatiotemporal consistency, sensor failures, near-field and far-field details, physical constraints, and decision-making risks into elements of the world state. This section discusses the next stage of open technical issues in sensor-based world models: robust multimodal perception, generalizable world-state representation, and decision-oriented evaluation and control, and proposes some possibilities for these open issues in long-tail scenarios.

### 11.1. Robust Multimodal Sensing

A fundamental challenge facing sensor-based world models is temporal and spatial inconsistency between heterogeneous sensors. Cameras, LiDAR, radar, IMUs, haptic arrays, and force sensors have different sampling rates, clocks, delays, and coordinate systems. Even small errors can lead to inconsistent state estimates, and these small errors are amplified in both far-field and near-field scenarios. For example, in the far-field scenarios of autonomous driving, asynchronous observations can lead to increasingly larger mismatches in position, geometry, and motion; in the near-field scenarios of robotics, they can result in direct errors in state estimation and action execution.

Potential solutions for long-tail scenarios could include the following. Instead of assuming perfectly synchronized sensor data streams, the model could implement dynamic routing conditionally based on the current scene, task, and sensor attributes. Simultaneously, training data could be randomly injected with various perturbations, such as clock drift and jitter, slow parameter drift or instantaneous jumps, and non-rigid cross-modal inconsistencies, reducing overly optimistic assessments of system robustness.

Another important issue is the robustness of models to missing or degraded modes. In real-world embodied systems, whether far-field or near-field, robotics or autonomous driving, any operating sensor is likely to experience performance degradation, especially if a critical sensor in a heterogeneous sensor suite fails, causing the model to lose important physical information.

Handling long-tail scenarios might involve treating missing modalities as special cases, but normal cases in actual deployment. For example, constructing a modality-agnostic state learning approach, i.e., degrading and reconstructing the world state representation from a subset of currently available sensors when sensor performance degrades or fails. Alternatively, modalities or multiple modalities could be randomly dropped during training; for instance, UniBEV [107] has used modality dropout for world models where cameras and LiDAR coexist.

### 11.2. Generalizable and Decision-Relevant World States

Meanwhile, a problem with state representation is the lack of a unified representation method. Current methods such as latent markers, BEV maps, occupancy fields, neural radiation fields, Gaussian distributions, object-centered graphs, and action conditional states have effectively characterized the diverse aspects of the physical world, but a world-state representation that simultaneously meets the perception, prediction, planning, and control needs of both far-field and near-field tasks is still lacking. For example, BEV and occupancy fields are effective for scene understanding in far-field space but cannot represent contact details in near-field space. Object-centered graph representations are effective for operation and relation reasoning tasks in the near field but lose the geometric details of the target object.

In long-tail scenarios, a promising direction is to explore generalizable world-state representation methods, constructing hierarchical, factorized, and multi-resolution abstract representations of world states, encompassing global spatial structure, target semantics, and local interactions. For example, BEVWorld [108] can achieve global low-resolution representations in far-field regions and fine-grained representations in high-risk near-field regions.

Many recent world-state models are built on compact latent states, which are often difficult to explicitly explain and verify. Furthermore, these latent states may fail to adjust predictive losses even when their representations do not align with stable physical states, leading to a cascading accumulation of small errors that ultimately result in severe consequences. Therefore, world-state learning should not only focus on short-term predictive accuracy but also consider the extent to the decision-related physical information retained within compact latent states.

In long-tail scenarios, we can continue to explore physically grounded and verifiable latent states. Combining explicitly encoded physical properties such as geometry with explicit physical constraints like dynamics in these latent states not only improves long-term prediction accuracy but also maps the model’s internal states to measurable physical variables. Incorporating supervision of varying intensities and performing verifiable, structured partitioning is designed for building physically interpretable world models [109].

### 11.3. Perception Metrics Versus Decision Quality

The mismatch between performance evaluation and decision quality in existing world models is one of the reasons for decision-making failures at the decision-making level. That is, high metrics do not directly lead to better planning and control. For example, in near-field scenarios of robot operation, small errors in the torso have little impact, while small errors in collision boundaries can lead to serious consequences. Future benchmarking and evaluation methods should not evaluate perception and prediction metrics in isolation, but rather test whether the learned world model can improve stability, safety, and robustness in the real physical world.

For example, in long-tail scenarios with pedestrians crossing, minor errors in vehicle exterior texture, non-road background, or large objects on either side typically do not interfere with driving decisions. Conversely, minor errors in critical areas such as lane boundaries, vehicle collision envelopes, and pedestrian positions can lead to incorrect driving decisions. In robotic operations, minor errors in the center position of a target object can be compensated for by gripper closure in grasping tasks; conversely, minor errors in contact boundaries, insertion gaps, or friction states in insertion tasks can lead to jamming or collision damage.

Future evaluation methods could incorporate decision sensitivity analysis. For instance, in autonomous driving, a small perturbation can be added to pedestrian position data; in robotic operations, the contact point position can be perturbed. If a small change in a state variable from a certain sensor causes the autonomous vehicle to switch from normal passage to emergency braking, or causes the robot to switch from stable insertion to jamming, then that variable is considered a critical state and should receive higher weight in world model training and evaluation.

### 11.4. Data, Simulation, and Benchmarking

Another open problem is the lack of standardized multimodal datasets and evaluation protocols for sensor-based world models. Driving datasets emphasize camera/LiDAR/radar/maps/trajectory labels, while robot manipulation datasets emphasize visual observations/proprioception/action trajectories. Tactile and force data are much less standardized, which limits the development of world models.

To cover long-tail events, future datasets can combine real, simulated, and generated data, and establish a hierarchical annotation system that connects the far and near fields. For example, autonomous driving data typically includes a global description of road structure but lacks near-field physical details such as minor scratches on the vehicle body; robot operation data usually contains fine-grained movements and contacts but lacks a large-scale description of scene structure. Therefore, it is possible to explore unifying the representation of data from multiple data sources and providing annotations of appropriate accuracy for different tasks. In addition, existing tactile sensors vary significantly in spatial resolution, range, surface materials, output formats, and calibration methods, necessitating further standardization of tactile and force data.

### 11.5. Mapping Design Recommendations to Evaluation Settings and Decision-Level Metrics

The recommendations above are only actionable when they are tied to a specific evaluation setting and to a decision-level metric that is sensitive to the failure mode they target. Table 15 summarizes this mapping for three representative SSD design recommendations. Each recommendation is paired with a primary evaluation setting (open-loop forecasting, closed-loop planning/control, or interactive simulation), a representative benchmark that supports that setting, and a decision-level metric whose value changes if the recommendation is violated. Uncertainty propagation and cross-scale feedback are treated together because both act on the same Decision Layer interface: they decide when a propagated state-level estimate should change the executed behavior.

To make these recommendations actionable, three measurement conventions are needed. First, the evaluation setting must use the same scene under both the recommended design and a matched baseline, so that the difference in the decision-level metric can be attributed to the design rather than to data variation. Second, the decision-level metric must be measured at the Decision Layer: perception metrics such as mAP, NDS, FVD, or reconstruction IoU inform state quality but cannot stand in for closed-loop planning success, collision rate, contact stability, or escalation rate. Third, both uncertainty propagation and cross-scale feedback share a single operational test. A perturbation is injected into either a latent state or a coarse parent state; if the system fails to escalate, replan, or downweight the action in time, the failure is recorded as a decision-level error, regardless of whether the State Layer reconstruction remained visually plausible. Implementing these conventions requires benchmarks that expose both sensor provenance and recovery protocols, and it is precisely this combination that the long-tail discussion in Section 12 identifies as the principal open gap.

## 12. Conclusions and Future Perspectives

To adapt to increasingly dynamic and complex real-world physical environments, effective embodied systems require embedding world models that account for both abstract scenarios and physical states. Sensors will no longer be merely edge data acquisition tools, but rather structural elements in physical-world modeling. Early embodied-system world models were typically based on visual input; recently, however, data from heterogeneous multimodal sensors such as LiDAR, RGB-D, audio, proprioception, and haptics have been explicitly incorporated.

Sensors serving world models need to position themselves as physical-intelligent infrastructure, that is, starting from sensor–model joint optimization mechanisms, designing novel multimodal sensors, calibrable far-field–near-field sensor arrays, low-latency tactile/force-feedback sensors, event-camera sensors, and learnable sensors, among others. However, compared to the rapid development of world models, current sensors and related physical devices cannot fully accommodate the long-tail physical constraints, far-field and near-field state representations, and cross-platform sharing of multi-layer representations that world models require. We organize the open challenges as follows:

1. Sensor data generation in long-tailed scenarios lacks physical constraints and reliability. Current simulations are better at generating images of long-tailed scenarios that “look realistic,” but this visual information conflicts with physical information such as tactile deformation, friction, and material response, lacking unified physical constraints and reliability [110]. Moreover, in long-tailed scenarios such as rain, snow, or low visibility, the data quality of cameras, radar, and LiDAR is directly affected.
2. Far-field and near-field data are not yet coupled through a continuous cross-scale state. Far-field and near-field sensors differ significantly across many physical parameters and currently maintain their own independent representations and control cycles. This separation produces no bidirectional update relationship between far-field prediction and near-field feedback. Near-field feedback is used only for instantaneous action correction and does not participate in the states and behaviors within the far-field model, causing subsequent planning to deviate continuously from the actual physical state. Conversely, near-field control cannot exploit far-field data to anticipate physical changes that are about to occur [111,112].
3. Cross-platform standardization is reflected only at the data format level. Existing cross-embodiment datasets can normalize some images, language, and end-effector actions, but the representation of sensor mounting location, field of view, range, latency, calibration status, and uncertainty remains coarse. For example, different robots have substantially different observation and action spaces, and current pipelines rely on coarse-grained alignment [94]. During cross-platform migration, high-level semantic representations remain biased by the raw sensor features at the lower layers.

This paper argues that sensor-based world-model construction should be understood as a system-level physical problem. The Sensor–State–Decision framework, serving as the system’s infrastructure, transforms heterogeneous physical information into actionable, unified representations that reflect the relationships among the three elements. On this basis, unified modeling of far-field and near-field perception provides a representation that supports environmental understanding and physical interaction simultaneously, replacing mere descriptions of static objects and structures in the environment. Such representations hold promise for advancing physical artificial intelligence in a constantly changing and uncertain physical world. In addition, heterogeneous sensors exhibit substantial variation across sensing distance, spatial resolution, signal-to-noise ratio, and other physical quantities. Therefore, beyond multimodal fusion of heterogeneous data, world models should also build a continuous far-field–near-field state representation that provides an interface to the sensor-centric world model. This interface enables far-field perception to set task objectives, motion boundaries, and safety constraints for near-field tasks, while near-field feedback corrects far-field state predictions in real time, moving from “multimodal fusion” to “cross-scale state coupling” and supporting global modeling within a continuous far-field–near-field representation.

As one research pathway for long-tailed scenarios, the Sensor–State–Decision framework treats perception, state representation, prediction, and behavior as a single continuous and interconnected model that subsumes reinforcement learning, vision–language–action models, world–action models, and digital twins. The sensor layer defines observable physical boundaries, such as the critical far-field information and near-field feedback required in long-tailed scenarios. The state layer translates observations from the sensor layer into internal states, particularly useful for reconstructing scene state variables under uncertainty. The decision layer then turns these state variables into actions, plans, or policies. New datasets and benchmarks will also emerge around the Sensor–State–Decision framework.

In summary, sensor-based world models connect the three elements of an embodied system: the physical environment, the internal state, and embodied actions. Through continuous far-field and near-field coupling and the Sensor–State–Decision framework, the drift of these three elements within the world model is corrected continuously, enabling migration and reuse across diverse AI physical platforms. Several open challenges remain, including the lack of unified physical laws to guide long-tail sensor data, persistent inconsistency between far-field and near-field data updates, and the absence of stable high-level semantic representations for cross-platform migration. Looking ahead, the sensor community should address these challenges through collaborative design that spans multi-source heterogeneous sensor devices, world models, and embodied platforms. Under the constraints of real-world deployment, a real-time feedback loop among the physical environment, internal state, and embodied actions will enable sensor-based world models to describe the physical world and to be continuously verified and safely acted upon through ongoing interactions.

## Figures and Tables

**Table 1:** A simplified comparison of sensor-related terminology in world model papers before and after 2023.

Period/Category	Representative Works	Explicit Use of “Sensor”	More Frequent Terms
Pre-2023 model-based reinforcement learning	World Models [1], PlaNet [2], Dreamer [3], MuZero [5]	Low	Observation, pixels, images, latent state, reward, value, policy
Pre-2023 autonomous driving simulation	SurfelGAN [11] and related driving simulation works	High	Camera, LiDAR, radar, sensor data, simulated sensor data
Post-2023 latent, spatial, and generative world models	DreamerV3 [4], V-JEPA 2.1 [12], Causal-JEPA [13], Genie [9], UniSim [14], WHAM [8]	Medium	Video, image, latent action, visual tokens, language action, spatial representation, generative decoder
Post-2023 robot world models	PointWorld [15], DreamZero [16], DreamGen [17], GR00T N1 [18], Visuo-Tactile World Models [19], DreamerV3 for microrobots [6]	Medium to High	Video, action, proprioception, RGB-D, 3D flow, tactile, force, robot state, trajectory
Post-2023 autonomous-driving world models	GAIA [20], DriveDreamer [21], DriveDreamer-2 [22], MUVO [23], Occ3D [24], UniDriveDreamer [25], Cosmos-Drive-Dreams [26], OmniDreams [27], accident anticipation world model [7]	High	Multi-sensor, camera, LiDAR, radar, ego state, trajectory, occupancy, HD map, bounding box, future observation
Post-2023 physical AI platforms and embodied systems	Cosmos [28], Cosmos 3 [29], DreamZero [16], DreamGen [17], GR00T N1 [18], Nature News Explainer [10]	High in a broad multimodal sense	Video, image, audio, action, robot data, fleet state, digital twin, synthetic trajectory, policy model

**Table 2:** A cross-domain comparison of sensor contributions to world models in robotics, autonomous driving, generative interactive environments, and physical AI.

Direction	Main Role of Sensors	What the World Model Needs to Predict	Representative Works
Robotics	Ground robot embodiment, contact dynamics, and action consequences	Object states, contact outcomes, and state changes caused by robot actions	PointWorld [15], DreamZero [16], DreamGen [17], GR00T N1 [18], Visuo-Tactile World Models [19], DreamerV3 for microrobots [6]
Autonomous Driving	Support multi-sensor perception, long-tail simulation, and safety evaluation	Traffic-agent behavior, ego-vehicle action consequences, and future multi-sensor observations	GAIA [20], DriveDreamer [21], DriveDreamer-2 [22], MUVO [23], Occ3D [24], UniDriveDreamer [25], Cosmos-Drive-Dreams [26], OmniDreams [27], accident anticipation world model [7]
Generative Interactive Environments	Convert visual experience into controllable state dynamics	The next visual state conditioned on user or agent actions	Genie [9], UniSim [14], WHAM [8]
Physical AI	Serve as multimodal interfaces for state estimation, synthetic data generation, digital twins, and closed-loop validation	How real physical systems evolve under different actions, environments, and sensing conditions	Cosmos [28], Cosmos 3 [29], DreamZero [16], DreamGen [17], GR00T N1 [18], Nature News Explainer [10]

**Table 3:** Data-extraction and coding scheme based on the Sensor–State–Decision (SSD) framework.

Dimension	Information Extracted	Representative Coding Values
Sensor modality	Observation source available to the system	Camera, LiDAR, radar, RGB-D, proprioception, tactile sensing, force/torque sensing, audio, and simulated observations.
Sensor properties	Physical and temporal characteristics of the observation process	Sensing range, field of view (FoV), spatial resolution, temporal resolution, sampling frequency, synchronization, calibration, and latency.
Sensor uncertainty	Sources of observation degradation or sensor unreliability	Measurement noise, occlusion, missing modality, asynchronous sampling, calibration error, sensor degradation, and sensor failure.
State representation	Form of the internal world-state representation	Latent state, bird’s-eye-view (BEV) representation, occupancy representation, geometric or neural-field representation, object-centric state, and interaction/contact state.
State composition	Types of information represented in the world state	Environmental state, agent state, and interaction state.
Transition/prediction model	Mechanism used to update or predict the world state over time	Recurrent state-space model (RSSM), recurrent neural model, transformer, diffusion model, autoregressive model, and physics-based dynamics.
Action conditioning	Whether agent actions or interventions explicitly affect predicted state evolution	Explicit, partial, or absent.
Decision interface	Downstream use of the current or predicted world state	State prediction, counterfactual reasoning, planning, policy learning, control, and simulation or synthetic data generation.
Uncertainty treatment	How uncertainty is represented or propagated toward downstream decisions	Explicit probability distribution or confidence, implicit stochastic representation, not reported, or not applicable.
Evaluation setting	Type of experimental or deployment validation	Open-loop evaluation, closed-loop evaluation, simulation, and physical-world deployment.
Operating regime	Characteristic spatial, temporal, and interaction scale of the SSD instance	Far-field, near-field, or hierarchical/multiscale SSD regime.
Application domain	Primary embodied-intelligence application	Autonomous driving, robotic manipulation, surgical robotics, generative interactive environments, and physical AI.

**Table 4:** Operational definitions and required mapping fields for the Sensor–State–Decision taxonomy.

SSD Layer	Operational Definition	Analogy	Required Fields
Sensor Layer	Measurement, sampling, simulation, or preprocessing interface that directly produces physical or simulated observations of the environment or embodied agent.	Input interface	Modality, range/FoV, spatial and temporal resolution, sampling rate, calibration, synchronization, quality/reliability, uncertainty, metadata.
State Layer	Explicit or implicit world-state representation built from current and historical observations to support prediction, planning, control, or decision-relevant inference.	Hidden representation	State type, temporal memory, geometry/BEV/occupancy/object-centric/scene graph/neural field/latent-token form, transition or prediction model, state uncertainty.
Decision Layer	Interface that uses current or predicted states to generate an action, plan, policy, trajectory, intervention, or decision-support output.	Output interface	Decision type, planning/control/policy/MPC/RL/language-conditioned action, horizon, control frequency, risk treatment, closed-loop output.

**Table 5:** Initial SSD mapping matrix for representative world-model systems and ambiguous cases.

System	Sensor Modalities	State Representation	Transition/Prediction	Decision Interface	Uncertainty Treatment	Evaluation Setting
World Models [1]	Simulated pixel observations	Latent visual code plus recurrent state	Recurrent rollout	Controller in imagination	Mostly implicit model uncertainty	Closed-loop simulation tasks
PlaNet/DreamerV3 [2,4]	Images or proprioception, task dependent	Recurrent state-space latent	Stochastic latent dynamics	Planning or actor-critic policy	Stochastic latent state	Simulated and embodied control
GAIA-1/DriveDreamer [20,21]	Camera-centric driving observations	Scene/video latent or structured condition	Future video or scene generation	No direct controller; planning support	Implicit generative uncertainty	Open-loop scenario evaluation
OccWorld [40]	Driving observations mapped to occupancy	3D occupancy world state	Future occupancy	No action execution; occupancy decision support	Occupied/free/unknown occupancy	Open-loop forecasting
Genie [9]	Internet video with inferred actions	Latent video/action tokens	Interactive video dynamics	Control in generated environment, no physical actuation	Implicit generative uncertainty	Interactive generated environments
Visuo-tactile WM [19]	Vision and tactile observations	Fused contact-rich latent state	Contact-aware rollout	Manipulation prediction and policy support	Contact ambiguity through multimodal prediction	Manipulation tasks

**Table 6:** Difference between SSD and related analytical frameworks.

Framework	Primary Focus	SSD Increment
POMDP	Formal partial observability, belief, action, and reward.	Adds explicit sensor provenance, reliability, spatiotemporal scale, and empirical layer interfaces.
State estimation	Recovering system state from observations.	Places estimation inside a sensor-to-state-to-decision chain and compares state abstractions by downstream use.
Model-based RL	Learning or using transition dynamics for planning and policy learning.	Tracks how real or simulated sensors form state and how metadata affect transfer, uncertainty, and decisions.
Perception–action loop	Closed-loop coupling between perception and action.	Inserts a comparable world-state interface, hierarchy, and uncertainty propagation path.
Digital twin pipeline	Synchronizing physical and digital representations.	Emphasizes observation provenance, abstraction, decision hierarchy, and sensor-conditioned world modeling.

**Table 7:** Operational terminology used in the SSD-based review.

Term	Operational Use in This Paper
World model	A learned or structured model that represents current world state and/or predicts future states or observations in a form supporting planning, control, policy learning, simulation, or decision support.
World state	An explicit or implicit observation-grounded representation that supports temporal prediction, environment representation, or decision conditioning; arbitrary visual features are not automatically world states.
Sensor-driven	A system whose observations have identifiable physical, simulated, or data-derived sensor provenance; video-only and web-video systems qualify only when provenance and interaction interface are identifiable.
Physical AI	Embodied or physically grounded AI systems that interact with, simulate, or make decisions about real or physically plausible environments.
Long-tail scenario	A low-frequency but high-consequence sensing, state, or decision condition, including rare observations, rare transitions, or rare risk-sensitive decisions.
Far-field/near-field	Relative operating regimes defined by sensing range, spatiotemporal resolution, interaction scale, uncertainty, task horizon, controllability, contact state, and decision granularity.

**Table 8:** Representative far-field sensor modalities in SSD.

Modality	Primary Contribution	Typical Uncertainty	SSD Relevance
Camera	Dense semantics and texture	Depth ambiguity, lighting, occlusion	Scene understanding and semantic state
LiDAR	Metric 3D geometry	Range sparsity, reflective surfaces	Occupancy, localization, 3D detection
Radar/4D radar	Range, velocity, adverse-weather sensing	Low angular resolution, noisy returns	Dynamic-state estimation and robust fusion
GNSS/IMU	Ego pose and motion	Drift, outage, synchronization error	State alignment and trajectory context

**Table 9:** Representative near-field sensor modalities in SSD.

Modality	Primary Contribution	Typical Uncertainty	SSD Relevance
Depth/wrist camera	Local geometry and object pose	Occlusion, calibration, viewpoint limits	Near-object state and manipulation context
IMU/encoder	Body and joint state	Drift, bias, actuator backlash	Action-conditioned state update
Tactile array	Contact geometry, texture, slip	Limited range, contact dependence	Contact-state prediction and grasp stability
Force/torque	Net wrench and load	Sensor bias, compliance ambiguity	Risk-aware control and force regulation

**Table 11:** Illustrative instantiations of recursive SSD levels across operating regimes. The numerical $\lambda_{\ell }$ and $\tau_{\ell }$ values are representative examples rather than classification thresholds.

Level and Operating Profile	Representative Sensor Layer $\theta_{\ell }$	State Layer	Decision Layer
Level 0: coarser/planning-oriented Broad context, coarser spatial state, longer task horizon, uncertainty dominated by partial observation and other agents, indirect controllability, contact usually absent, and comparatively lower control frequency. Illustrative: $\lambda_{\ell }∼$ 10–200 m in autonomous driving or room scale in robotics; $\tau_{\ell }∼$ 0.1–1 s.	Representative exteroceptive suites, for example camera–LiDAR–radar stacks [31,50,53]	BEV grids, occupancy volumes, neural fields [31,40,63]	Trajectory-level planning and scene prediction [21,61]
Level 1: object/skill-oriented More local context, finer spatial state, shorter horizon, stronger direct controllability, intermittent or anticipated contact, and faster updates. Illustrative: $\lambda_{\ell }∼$ 1 m; $\tau_{\ell }∼$ 0.1 s.	Close-range vision with proprioceptive integration [55,56,57]	Object-centric and action-conditioned latent states [64,65,66,71]	Skill- and grasp-level sub-goals via latent imagination [3,71]
Level 2: fine/contact-oriented Local high-resolution state, short decision timescale, direct controllability, contact-state dependence, and high-frequency closed-loop updates. Illustrative: $\lambda_{\ell }∼$ mm–cm; $\tau_{\ell }∼$ 1–10 ms.	Tactile arrays, force/torque sensing, and high-rate proprioception [19,48,58]	Contact-state latent representations [19]	Force- and torque-level closed-loop control [4,57]

**Table 12:** SSD-oriented capability matrix for representative datasets and benchmarks.

Dataset/Benchmark	Domain	Sensor and Temporal Structure	State/Action Annotations	SSD Regime	Long-Tail Coverage	Closed-Loop Support	Best-Suited Research Question
nuScenes [50]	Autonomous driving	Synchronized surround cameras, LiDAR, radar, GPS/IMU, and temporally ordered urban scenes	3D boxes, tracks, HD maps, ego state, and trajectories	Far-field	Broad urban variation, but not primarily a rare-event benchmark	No; recorded logs	How should heterogeneous sensors be fused into BEV, occupancy, tracking, and forecasting states?
nuPlan [81]	Autonomous driving	Driving logs with camera, LiDAR, vehicle state, maps, and temporal trajectories	Tracks, ego state, plans, and driving trajectories	Far-field	Scenario diversity is useful, but long-tail coverage is not the primary design target	Yes; planning-oriented closed-loop evaluation	Does a predicted world state support safe trajectory generation and closed-loop planning?
K-Radar [53]	Autonomous driving	4D radar, LiDAR, cameras, GPS/IMU under varied weather	3D boxes and multimodal geometric observations	Far-field	Strong adverse-weather coverage	No; recorded logs	How robust is Sensor-to-State estimation when optical modalities degrade?
RLBench [93]	Robot manipulation	Simulated RGB-D observations, masks, robot state, language, and temporally ordered task execution	Robot states, actions, demonstrations, and task definitions	Mesoscopic to near-field	Broad task and object variation rather than direct rare-event collection	Yes; interactive simulator	Can an action-conditioned state support planning, imitation, and multi-step manipulation?
Open X-Embodiment [94]	Robot manipulation	Cross-robot visual and proprioceptive trajectories aggregated from many embodiments	Standardized state, action, trajectory, and instruction data	Mesoscopic	Breadth-driven generalization across tasks, platforms, and embodiments	No; offline dataset	Which state and action representations transfer across heterogeneous robot embodiments?
RH20T [95]	Robot manipulation	Multi-view RGB, force, audio, robot state, and action trajectories	Robot state, actions, language, force, and contact-rich task information	Mesoscopic to near-field	Broad contact and task diversity; not a dedicated long-tail benchmark	No; recorded trajectories	How should proprioception, force, audio, and vision be combined for contact-aware state prediction?
TaF-Dataset [73]	Tactile/force interaction	Synchronized tactile observations with six-axis force/torque measurements	Spatial force maps and contact-related measurements	Near-field	Not designed primarily for long-tail evaluation	No; recorded interactions	How accurately can tactile observations be aligned with force and contact-state representations?
SurgSync [96]	Surgical robotics	Synchronized stereo video, external cameras, dVRK kinematics, and contact sensing	Kinematic state and contact information linked to surgical actions	Near-field	Not primarily a long-tail benchmark	No; recorded procedures	How can visual, kinematic, and contact observations support surgical state estimation and action prediction?
CODA [97]	Autonomous driving	Camera and LiDAR observations centered on corner cases	Rare-object and corner-case annotations	Far-field	Direct long-tail coverage	No; recorded cases	How does perception and state construction fail on rare objects outside common driving distributions?
BEHAVIOR-1K [98]	Robot manipulation	Simulated household environments with visual observations, object states, and physics	Object states, actions, task definitions, and state transitions	Mesoscopic to near-field	Large task and scene diversity supports generalization testing	Yes; interactive simulation	Can a world model support long-horizon state transitions and closed-loop household planning?

**Table 14:** Representative datasets and benchmarks for robot manipulation world-model research.

Dataset	Main Data and Annotations	Representative Research Use Cases
RoboNet [103]	Multi-robot camera trajectories with actions, objects, and platform variation.	Video prediction, visual foresight, inverse dynamics, and cross-robot transfer.
RLBench [93]	Simulated tasks with RGB-D, masks, robot states, language, actions, and demonstrations.	Imitation learning, multitask learning, few-shot adaptation, and planning.
RoboMimic [104]	Simulated and real tasks with RGB, robot states, actions, and human demonstrations.	Offline imitation learning, offline RL, and demonstration-quality analysis.
BridgeData V2 [105]	Real-robot trajectories with RGB, states, actions, goals, language, and diverse scenes.	Goal-conditioned learning, language-conditioned control, and environment generalization.
Open X-Embodiment [94]	Standardized visual, state, action, and instruction data across robot embodiments.	Cross-embodiment pretraining, generalist policies, and cross-platform transfer.
DROID [106]	In-the-wild multi-view RGB trajectories with states, actions, language, and diverse tasks.	Real-world imitation learning, policy pretraining, and cross-scene generalization.
RH20T [95]	Contact-rich trajectories with RGB, force, audio, states, actions, and language.	Multimodal fusion, contact-aware modeling, one-shot learning, and state prediction.

**Table 15:** Mapping of representative SSD design recommendations to evaluation settings and decision-level metrics.

SSD Design Recommendation	Evaluation Setting	Representative Benchmark	Decision-Level Metric
Dynamic routing under temporal/spatial sensor inconsistency (Sensor Layer)	Closed-loop planning and control with injected clock drift, jitter, modality dropout, and asynchronous multimodal logs	nuPlan [81], K-Radar [53]	Planning success rate, collision rate, and route-completion time under injected synchronization and modality-dropout perturbations
Hierarchical and multi-resolution state representation (State Layer)	Interactive closed-loop simulation in far- and near-field regimes combined with open-loop reconstruction quality on the same scene	RLBench [93], ManiSkill2 [82], nuScenes [50]	Task success rate, contact-aware insertion/gouge-free completion, and IoU or mAP gap between coarse and fine regions under identical sensor input
Uncertainty propagation and cross-scale feedback through result reporting (Decision Layer)	Closed-loop evaluation with adversarially perturbed latent or coarse states, paired with rare-event replay	nuPlan [81], Open X-Embodiment [94]	Escalation rate to the parent level, recovery time after a high-uncertainty observation, and negative log-likelihood or expected calibration error of the chosen action versus the realized outcome

## Data Availability

No new data were created or analyzed in this study. All information reviewed in this article is available in the cited publications and publicly available datasets. Data sharing is not applicable to this article.
